# Identification of target genes for wild type and truncated HMGA2 in mesenchymal stem-like cells

**DOI:** 10.1186/1471-2407-10-329

**Published:** 2010-06-25

**Authors:** Jørn Henriksen, Marianne Stabell, Leonardo A Meza-Zepeda, Silje AU Lauvrak, Moustapha Kassem, Ola Myklebost

**Affiliations:** 1 CAST, Cancer Stem Cell Innovation Centre, Department of Tumor Biology, The Norwegian Radium Hospital, Oslo University Hospital, N-0027 Oslo, Norway; 2 Department of Molecular Bioscience, University of Oslo, Oslo, Norway; 3 Department of Endocrinology, University Hospital of Odense, Denmark & Stem Cell Unit, College of Medicine, King Saud University, Riyadh, KSA

## Abstract

**Background:**

The HMGA2 gene, coding for an architectural transcription factor involved in mesenchymal embryogenesis, is frequently deranged by translocation and/or amplification in mesenchymal tumours, generally leading to over-expression of shortened transcripts and a truncated protein.

**Methods:**

To identify pathways that are affected by sarcoma-associated variants of HMGA2, we have over-expressed wild type and truncated HMGA2 protein in an immortalized mesenchymal stem-like cell (MSC) line, and investigated the localisation of these proteins and their effects on differentiation and gene expression patterns.

**Results:**

Over-expression of both transgenes blocked adipogenic differentiation of these cells, and microarray analysis revealed clear changes in gene expression patterns, more pronounced for the truncated protein. Most of the genes that showed altered expression in the HMGA2-overexpressing cells fell into the group of NF-κB-target genes, suggesting a central role for HMGA2 in this pathway. Of particular interest was the pronounced up-regulation of SSX1, already implicated in mesenchymal oncogenesis and stem cell functions, only in cells expressing the truncated protein. Furthermore, over-expression of both HMGA2 forms was associated with a strong repression of the epithelial marker CD24, consistent with the reported low level of CD24 in cancer stem cells.

**Conclusions:**

We conclude that the c-terminal part of HMGA2 has important functions at least in mesenchymal cells, and the changes in gene expression resulting from overexpressing a protein lacking this domain may add to the malignant potential of sarcomas.

## Background

HMGA2 belongs to a family of nuclear proteins that contain an N-terminal part that recognizes and binds to AT-rich regions in the DNA [1] and an acidic C-terminal tail that probably modulates their interactions with DNA [2], and proteins [3]. HMGA proteins are not transactivators on their own, but modulate the assembly of transcriptional complexes at various levels [4-8], and may also regulate gene transcription through protein-protein interactions without direct contact with DNA [9-11] or by altering chromatin structure [12-16]. Interestingly, a recent study suggested that both HMGA1 and HMGA2 are taking active parts in the formation of senescence-associated heterochromatin foci and maintenance of the growth-arrested state [17,18].

HMGA2 is expressed during embryonic development while it is almost undetected in normal adult tissues [19,20]. HMGA2 plays a critical role early in adipogenesis, probably by regulating the proliferation of preadipocytes during differentiation [21], and mice lacking functional HMGA2 protein exhibit a *pygmy *phenotype with drastic reduction of adipose tissue [22]. On the other hand, over-expression of a truncated HMGA2 protein lacking the acidic C-terminal tail results in a giant phenotype with multiple lipomas [23,24].

Aberrations in the chromosomal region 12q14-15 that affect *HMGA2 *are frequent in a variety of tumours. In benign tumours of mesenchymal origin, *HMGA2 *is often rearranged by translocation, and the resulting chimeric transcripts are formed by fusion of the DNA-binding domains, coded by exons 1-3, to ectopic sequences [25-27]. In sarcomas, *HMGA2 *is frequently and selectively amplified and rearranged [28]. We have cloned and sequenced a number of these cancer-associated ectopic sequences from 12q as well as other chromosomes [29]. The only common factor that was found was the loss of the 3'-untranslated region (UTR) as well as the last two exons, resulting in fusion proteins containing as little as one extra amino acid replacing the C-terminal part [29].

Recently it was shown that the HMGA2 3'-UTR contains target sites for the *let-7 *miRNA, and thus the above mentioned rearrangements lead to increased levels of *HMGA2 *protein due to loss of miRNA-mediated repression [30,31]. Thus the attention has shifted from possible oncogenic effects of loss of the acidic domain to effects of increased expression, even of the wild type protein. Furthermore, it turns out that the balance between *let-7 *and HMGA2 governs the exit of cells from the undifferentiated and self-renewing state, and HMGA2 is now thought to be central in cancer in general [32-35]. Because HMGA2 most frequently is rearranged in well-differentiated liposarcomas, border-line tumours resembling adipose tissue, most sarcoma cell lines, representing highly malignant cancers with a different tissue type, would not be appropriate to detect a phenotype when the gene is over-expressed. We therefore chose an immature, stem-like mesenchymal cell line with adipogenic potential to investigate whether the wild type and truncated forms of the protein activate the same pathways, by performing gene expression analysis.

## Methods

### DNA constructs

The coding sequence and the part of the HMGA2 gene encoding the DNA-binding domain (exon 1-3) were PCR-amplified from pBluescript-HMGI-C [27] and cloned into pEGFP-C3 (Clontech, Palo Alto, CA, USA) generating pEGFP-HMGA2_WT _and pEGFP-HMGA2_TRUNC_, respectively.

### Cell culture and stable transfectants

The human mesenchymal stem cell line (hMSC) transduced with human telomerase reverse transcriptase (hTERT), hMSC-TERT20 cells [36,37] were transfected with the HMGA2 constructs described above, using Fugene (Roche Diagnostics, Basel, Switzerland) according to the manufacturer's protocol. Stable clones were selected with 400 μg/ml G418 (Gibco BRL, Gaithersburg, MD, USA) and clones with strong GFP-fluorescence were established using fluorescence activated cell sorting. Cells were maintained in RPMI1640 (Gibco) supplemented with 10% fetal bovine serum and 100 μg/ml G418.

### Fluorescence microscopy

Transfected hMSC-TERT20 cells grown on coverslips were fixed with 10% formalin solution (Sigma-Aldrich, St. Louis, MO, USA), permabilized with 0.1% Triton X-100 in PBS and blocked with 5% FCS. Staining for HMGA2 and B23 (nucleophosmin - a marker for the nuclear matrix)[38] was performed using rabbit-anti HMGA2 (Strategic Diagnostics Inc., Newark, DE, USA), and mouse-anti B23 followed by DyLight549-conjugated donkey anti-rabbit and DyLight649-conjugated donkey anti-mouse antibodies (Jackson Immunoresearch Laboratories, West Grove, PA, USA), respectively. The samples were mounted in Prolong Gold antifade reagent with DAPI (Invitrogen, Carlsbad, CA, USA). The fluorescence was visualized using a Zeiss LSM 510 confocal microscope and pictures were taken of thin single plane sections.

### Differentiation assay

The hMSC-TERT20 cells as described above, were seeded at 10 000 cells/cm^2 ^in medium supplemented with a cocktail of 0.5 mM methylisobutylxanthine (Sigma-Aldrich, St. Louis, MO, USA ), 1 μM dexamethasone (Decadron™, MSD, Haarlem, Nederland), 2 μM insulin (Sigma-Aldrich) and 20 μM Rosiglitazone (GlaxoSmithKline, Harlow, UK) for 14 days. To monitor the differentiation process, cultures were stained with Oil Red O (Serva, Heidelberg, Germany). The stained neutral lipids were extracted and the absorbance at 490 nm measured. The number of cells was estimated by using SRB protein assay on the same cultures. Briefly, proteins were precipitated with 10% trichloracetic acid (Merck, Darmstadt, Germany) and assayed by staining with sulforhodamine B (Sigma-Aldrich), extraction and measuring the dye absorbance at 540 nm [39].

### Northern blot analysis and quantitative RT-PCR

Total RNA was isolated from sub-confluent growing cells using Trizol reagent (Invitrogen) according to the manufacturer's instructions. For Northern blot analysis, the samples of RNA (10 μg) were run on a 1% formaldehyde agarose gel, blotted onto a positively charged nylon membrane and hybridizations were performed as described [40]. Probes were sequentially hybridized to the membrane and signals from an 18S rRNA oligonucleotide were used to correct for unequal sample loading.

For quantitative RT-PCR, 0.5 μg of RNA from each sample was reverse transcribed using the High-Capacity cDNA Archive Kit (Applied Biosystems, Foster City, CA, USA) according to the manufacturer's instructions. The resulting cDNAs were used for real-time PCR using TaqMan Gene Expression Assays (Applied BioSystem; Additional file 1 Table S1). Threshold cycle (C_t_) values were measured and the comparative C_t _method [41] was used to calculate the log_2- _fold difference in transcript level of the over-expressing cell lines relative to the parental. See Additional file 1 Table S1 for probes.

### Western blot analysis

Cells were harvested and protein lysates were prepared by sonicating cells resuspended in a nuclear lysis buffer of 50 mM Tris-HCl, pH 7.5, 0.1% SDS, 0.5 M NaCl, 1% NP-40, 1% DOC, 2 mM EDTA supplemented with protease inhibitors (all from Sigma-Aldrich). 5 μg protein were subjected to SDS-PAGE, transferred onto an Immobilon-P membrane (Millipore, Billerica, MA, USA) and probed with anti-HMGA2 (Santa Cruz Technologies, Santa Cruz, CA, USA) and anti-tubulin (Santa Cruz). Secondary antibodies conjugated to horse radish peroxidase (Dako, Glostrup, Denmark) were used according to the manufacturer's instructions. Detection of antibody signals was performed with chemiluminescence using SuperSignal West Dura Extended Duration Substrate (Pierce, Rockford, IL, USA).

### Microarray analysis

Total RNA was isolated from sub-confluent growing cells using Trizol reagent according to the manufacturer's protocol and global expression analysis was performed on HG-U133 Plus 2.0 oligonucleotide arrays (Affymetrix, Santa Clara, CA, USA). Labelling, hybridisation and scanning were performed at the Rikshospitalet University of Oslo Microarray Core Facility http://microarray.rikshospitalet.no using standard conditions. As we aimed to detect changes above the variation expected from different sub-lines, i.e. above 3-fold, one array per sample was sufficient (the technical variation between arrays being much smaller).

### Flow cytometry analysis of surface markers

To verify differential expression of CD24 and HLA-DR on the cell surface, subconfluent growing cells were harvested, incubated with phycoerythrine-conjugated antibodies and rinsed several times in PBS. IFN*γ*-treated cells were cultured with 100 U/ml INF*γ *(Sigma) for 40 hours prior to antibody staining. Antibody staining was detected with a triple-focus DIVA flow cytometer (Becton Dickinson, Franklin Lakes, NJ, USA). Antibodies used; anti-HLA-DR (Becton Dickinson); anti-CD24 (Beckman-Coulter, Fullerton, CA, USA). Data were analyzed with FlowJo software (Tree star, Ashland, Oregon, USA).

### Analysis of promoter sequences

Analysis of putative transcription factor binding sites was performed using MatInspector http://www.genomatix.de/cgi-bin/matinspector/matinspector.pl and TESS http://www.cbil.upenn.edu/tess/. The genomic DNA sequences were retrieved from the Ensembl genome browser http://www.ensembl.org/index.html covering 1500 base pairs of the 5' flanking regions (upstream from the transcriptional start sites) and 500 base pairs downstream. A core similarity of at least 0.80 from MatInspector or TESS analysis was used as cut-off for potential match to known transcription factor recognition sequences.

### Microarray data analysis

For GeneChip arrays, the raw intensity of individual samples was normalized using Bioconductor and the GC-RMA algorithm http://www.bioconductor.org[42]. Normalized gene expression levels for each transfected cell line were compared with the parental cell line and probe sets that exhibited less than 3-fold intensity change were removed from further analysis. The remaining probe sets were initially annotated according to entries in the NetAffx database http://www.affymetrix.com. Using the Database for Annotation, Visualization and Integrated Discovery (DAVID located at http://apps1.niaid.nih.gov/david[43], lists of differentially expressed genes, common or specific for the samples, were analysed to identify Gene Ontology (GO) terms that were significantly overrepresented using a modified Fisher Exact test (EASE-score < 0.05). The redundancy in the annotations was reduced by applying the clustering algorithm available in DAVID using intermediate stringency and default settings for the Kappa statistics.

## Results

### Exogenous HMGA2 is localized to the nucleus

**Figure 1:**
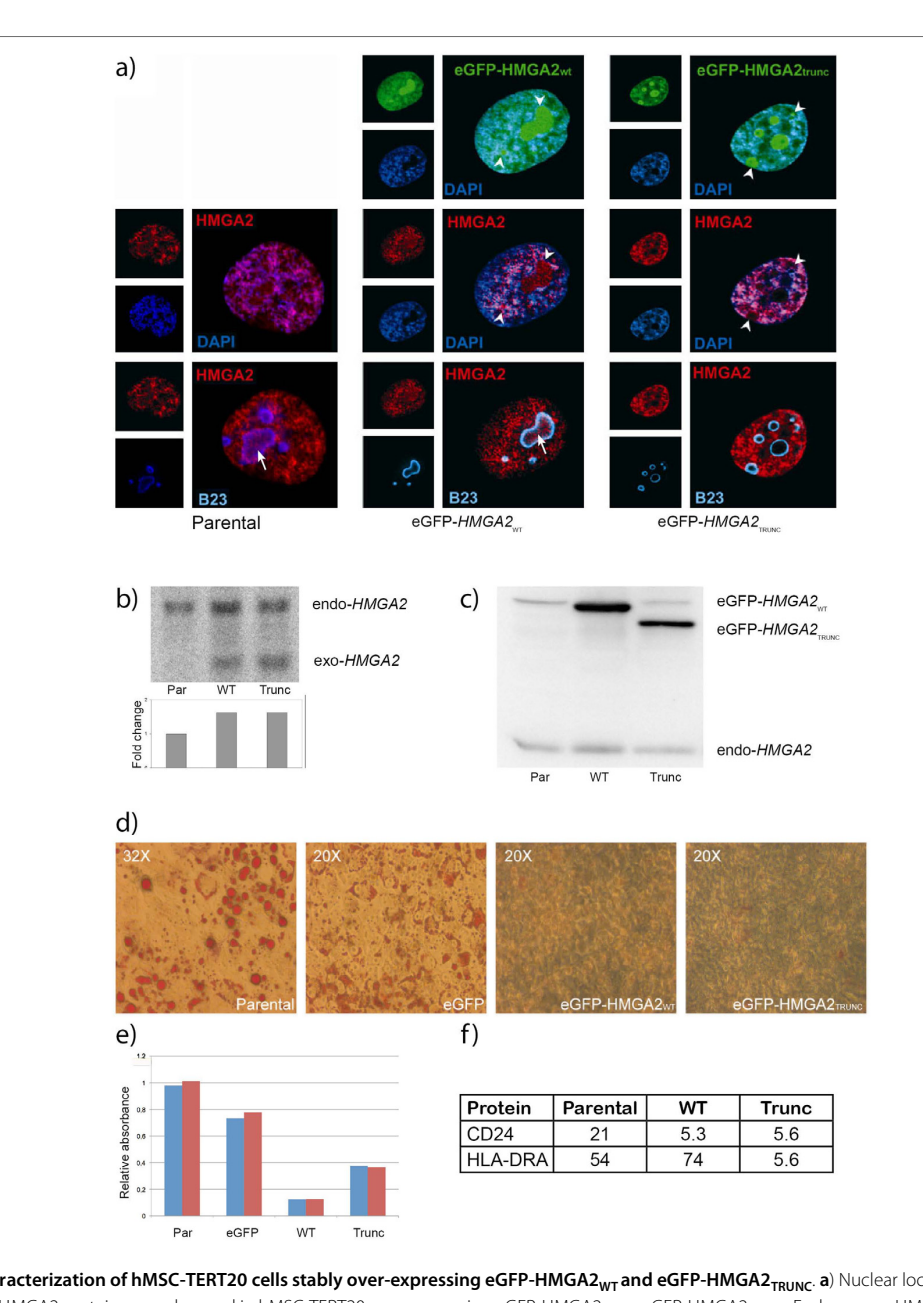
**Characterization of hMSC-TERT20 cells stably over-expressing eGFP-HMGA2_WT _and eGFP-HMGA2_TRUNC_**. **a**) Nuclear localization of eGFP-tagged HMGA2 proteins was observed in hMSC-TERT20 over-expressing eGFP-HMGA2_WT _or eGFP-HMGA2_TRUNC_. Endogenous HMGA2 was visualized by immunofluorescent staining with an anti-HMGA2 antibody (The intensity in parental cells was adjusted to make observation of nucleolar fluorescence possible). The nucleolus was detected with an anti-B23 antibody, while heterochromatin was stained by DAPI, showing co-localization of HMGA2 with both nucleoli and heterochromatin. Arrowheads show concentration of endogenous or eGFP-tagged HMGA2 at sites that correspond with nucleoli, while an arrow indicates discrete foci of HMGA2 within nucleoli. **b**) Expression levels of endogenous and exogenous *HMGA2 *transcripts; expected size of the endogenous transcript is 4468 bp, while the exogenous is only 327 bp due to the lack of the 3' UTR. Top panel, northern blot of HMGA2 mRNAs. Lower panel, quantitation of total *HMGA2 *mRNA levels by real-time PCR, normalized for TBP expression and represented as fold induction over parental cells. **c**) Expression levels of endogenous and exogenous HMGA2 proteins, as detected on western blot. **d**) Accumulation of fat in cell cultures grown in basal medium supplemented with MDI and Rosiglitazone for 14 days. **e**) Relative accumulation of fat-bound Oil Red O after 14 days of differentiation of hMSC-TERT20 cells. Data from two independent experiments are shown. Par, Parental cells; eGFP, eGFP-transduced; WT, transduced with eGFP-HMGA2_WT_; Trunc, transduced with eGFP-HMGA2_TRUNC _f) Expression of CD24 and HLA-DRA on each hMSC TERT20-derived cell lines as measured by flow cytometric mean fluorescence intensity (MFI).

To be able to monitor localization of the proteins, an enhanced green fluorescence protein (eGFP) tag was fused to the amino terminal end of both HMGA2 proteins. Cells expressing eGFP-HMGA2_WT _or eGFP-HMGA2_TRUNC _accumulate the eGFP-tagged proteins in the nuclei, especially in DAPI-dense and nucleolar regions (Fig. 1a), whereas eGFP alone showed general fluorescence throughout the cell, with lower level in nucleoli than in the rest of the nuclei (not shown). Noteworthy, both endogenous and fusion proteins co-localized with DAPI-dense heterochromatin but they were also present in numerous discrete foci that did not stain well by DAPI. Moreover, both fusion proteins appeared to be enriched in the nucleoli, detected by immunostaining against the nuclear matrix marker nucleophosmin/B23 [38]. Immunostaining for HMGA2, on the other hand, gave a more even fluorescence also covering the nucleoli for both endogeneous and ectopic HMGA2, and it is unclear whether this indicates that the apparent nucleolar enrichment of eGFP fusion proteins was a result of overexpression, or reduced nucleolar immunostaining could be due to incomplete penetrance of antibodies into the nucleoli compared to the efficient fluorescence from the fluorescent eGFP tags. Interestingly, endogenous HMGA2 seemed to be distributed throughout the granular region in the nucleolus whereas the granular nucleophosmin/B23 was restricted to the periphery. In addition, the fusion proteins stayed bound to the condensed chromosomes during mitosis (data not shown).

The endogenous *HMGA2 *transcript and protein was readily detected in hMSC-TERT20 cells (Fig. 1b and 1c), and the ectopic transcript showed a lower steady-state level than the endogenous transcript, as less than two-fold increase of HMGA2 transcripts was measured by quantitative PCR in the over-expressing cell lines. However, the levels of ectopically expressed protein was increased several fold relative to endogenous HMGA2.

### HMGA2 abolishes differentiation into the adipocytic lineage

Compared to the parental cells, forced expression of full-length or truncated HMGA2 abolished the ability of the cells to differentiate towards adipocytes and accumulate fat (Fig. 1d). Noteworthy, the growth of the HMGA2-overexpressing cells was unaffected by addition of differentiation medium, whereas the proliferation of the parental cells was inhibited when differentiation was induced. A control cell line expressing eGFP only showed growth and differentiation patterns similar to the parental line (i.e. within the variation expected when different passages of these cells was used, Fig 1d). The proliferation of the different transfectants was indistinguish¬able from that of the parental cells in the absence of differentiation (not shown).

### Identification of transcriptional targets

To find out how HMGA2 affects transcription, and possible effects of truncation, we analyzed the gene expression profiles of proliferating cultures of the over-expressing and the parental cell lines using the HG-U133 Plus 2.0 Affymetrix GeneChips. We identified 408 transcripts that were up-regulated more than 3-fold relative to the recipient cells in either of the two HMGA2-expressing lines, while 477 transcripts were down-regulated more than 3-fold. The microarray data were deposited in the Array Express public database http://www.ebi.ac.uk/microarray-as/ae/, accession number E-MEXP-2647), and a list of the most differentially expressed genes is shown in Table 1 (the complete list of transcripts changed more than 3-fold is given in Additional file 2 Table S2). Although the microarray technique can be used to detect much smaller changes, we chose 3-fold as a level where we would avoid smaller differences due to the prolonged separate culturing of each transfectant line.

**Table 1:** Identification of genes most differentially expressed between the parental cell line and the over-expressing cell lines

		**Changes in HMGA2**_**WT**_**cells**					Changes in HMGA2trunc cells				
	**Rank**	**Gene**	**Description**	**MA**	**qPCR**	Rel. to EGFP*	**Gene**	**Description**	**MA**	**qPCR**	Rel. to EGFP*

**a)**	**1**	*CXCL6*	Chemokine (C-X-C motif) ligand 6	-8.4	-6.7	-2.2	*CXCL6*	Chemokine (C-X-C motif) ligand 6	-9.0	-6.3	-2.2
	**2**	*IL8*	Interleukin 8	-7.5	-7.1	-6.4	*HLA-DRA*	Major histocompatibility complex class II antigen DRA	-8.7	-8.1	-7.2
	**3**	*CD24*	CD24 molecule	-7.1			*HLA-DPA1*	Major histocompatibility complex class II antigen DPA1	-8.0	-6.8	-5.5
	**4**	*C13orf33*	Chromosome 13 open reading frame 33	-6.5			*ChGn*	Chondroitin beta-1,4-N-acetylgalactosaminyltransferase	-6.5		
	**5**	*PTGES*	Prostaglandin E synthase	-6.3			*PKIB*	cAMP-dependent protein kinase inhibitor 2	-6,4	-4.9	-5.0
	**6**	*ChGn*	Chondroitin beta-1,4-N-acetylgalactosaminyltransferase	-5.6			*IFI44L*	Interferon-induced protein 44-like	-6.2		
	**7**	*IL6*	Interleukin 6	-5.5			*LAMA4*	Laminin alpha chain	-6.2		
	**8**	*PDE4D*	cAMP-specific phosphodiesterase 4D	-5.2			*SLC38A5*	Solute carrier family 38, member 5	-6.0		
	**9**	*IL1RN*	Interleukin 1 receptor antagonist	-5.1	-4.4	-3.0	*PTPRN2*	Protein tyrosine phosphatise, receptor type, N2	-5.5		
	**10**	*CES1*	Carboxylesterase 1	-5.1			*C13orf33*	Chromosome 13 open reading frame 33	-5.4		
	**11**	*KIAA1644*	KIAA1644 protein	-5.0	-3.4	-1.7	n/a	Similar to Wnt-1 inducible signalling pathway protein 1	-5.4		
	**12**	*SAMSN1*	SH3-SAM adaptor protein	-4.8			*IL1B*	Interleukin1 beta	-5.3	-4.3	-2.2
	**13**	*SERPIND1*	Serpin peptidase inhibitor, clade D, member 1	-4.7			*F2RL1*	Coagulation factor II receptor-like 1	-5.2		
	**14**	*FAM5C*	Familiy with sequence similarity 5,member C	-4.4			*CD74*	CD74 antigen-associated invariant chain	-5.2	-5.4	-4.3
	**15**	n/a	Transcribed sequence FLJ 26764	-4.3			*IL1RN*	Interleukin 1 receptor antagonist	-5.2	-5.2	-3.8

**b)**	**1**	*ZBED2*	Zinc finger BED domain-containing protein 2	5.3	4.5		n/a	transcribed sequence FLJ33010	6.7		
	**2**	*SHROOM2*	Shroom family member 2	4.6			*CXCL12*	Chemokine (C-X-C motif) ligand 12	6.4	2.7	2.0
	**3**	*FGF13*	Fibroblast growth factor 13	4.5	4.6	5.6	*PRSS7*	Serine protease 7	6.0		
	**4**	*BAI3*	Brain-specific angiogenesis inhibitor 3	4.5	4.4	5.4	*SULT1B1*	Sulfotransferase 1B1	5.8		
	**5**	*PLXNA4*	Plexin A4	4.4			*SSX1*	Synovial sarcoma, X breakpoint 1	4.9	9.6	10.2
	**6**	*LCP1*	Lymphocyte cytosolic protein 1	4.1	3.9	2.1	*MMP3*	Matrix metalloproteinase-3	4.7		
	**7**	*C20orf197*	Chromosome 20 open reading frame 197	4.0			*CYB5R2*	Cytochrome b5 reductase 2	4.5		
	**8**	*G0S2*	G0/S switch 2	3.7	5.6	5.4	*KISS1*	KiSS-1 metastasis-supressor	4.5		
	**9**	*HDAC9*	Histone deacetylase 9	3.7	1.5		*EDN1*	Endothelin 1	4.5		
	**10**	*SIGLEC15*	Sialic acid binding Ig-like lectin 15	3.7			*SYNE1*	Synaptic nuclear envelope protein 1	4.4		
	**11**	*HCLS1*	Hematopoietic cell-specific Lyn substrate 1	3.6	2.5	3.6	*HAPLN1*	Hyaluronan and proteoglycan link protein 1	4.4		
	**12**	*LOC646340*	similar to Ankyrin repeat domain-containing protein 18A	3.6			n/a	transcribed sequence FLJ35091	4.4		
	**13**	*GATA3*	GATA binding protein 3	3.5	3.6	>10	n/a	Similar to TATA box-binding protein-associated factor 1B	4.3		
	**14**	*SH3BGRL2*	SH3 domain binding glutamic acid-rich protein like 2	3.5			*BAI3*	Brain-specific angiogenesis inhibitor 3	4.2	4.6	5.6
	**15**	*PDE4B*	cAMP-specific phospodiesterase 4B	3.5			n/a	Similar to cytochrome c oxidase I	4.1		

The 15 most down- (a) and up-(b) regulated genes were ranked according to the expression fold change between transfectants and parental cells based on the microarray data. Changes in gene expression were validated by real time PCR using TaqMan assays, values in brackets are relative to those for the eGFP controls, for comparison. The expression level of each target gene was normalized for TBP expression and represented as fold induction over parental cells. Fold change ratios from both microarray (MA) and real-time assay (qPCR), including qPCR values (marked by an asterisk) calculated relative to those from eGFP only control cells, are shown.

Interestingly, although both protein variants similarly affected many genes, a large number of genes were differently affected. Out of 408 genes that showed up-regulation, 66 genes were up regulated by both full-length and truncated HMGA2. In addition, 84 and 258 were up-regulated in the cells expressing full-length and truncated HMGA2 respectively. On the other hand, 139 genes were down-regulated by both proteins while 99 and 239 were down-regulated by full-length and truncated HMGA2 respectively. As the cells were analysed in their undifferentiated state, we could not monitor genes directly involved in differentiation. Although we detected no change of the known HMGA2 target *Imp2 *(*IGF2BP2*) [44] in this system, the about 2-fold down regulation by both protein variants of both PPARα and PPARγ, central regulators of adipogenesis, could contribute to the differentiation block. In near all cases quantitative real-time PCR (qPCR) analyses confirmed the direction of changes in transcript levels observed by microarrays (Table 1). An exception was members of the SSX gene family, which were poorly distinguished by the microarray probes, and of which only SSX1 was confirmed by real-time PCR. Discrepant results were also found for *LCP1*, *FGF1 *and *HDAC9*, which most likely were caused by alternative splicing according to entries in the Alternative Splicing and Transcript Diversity database http://www.ebi.ac.uk/astd/main.html.

The over all correlation between microarray and real-time ratios between HMGA2 transfectants and parental cells was 0,96. The cells transfected with eGFP only were included in the real-time validation as indicated in Table 1, and although there were some discrepancies, the over all correlation between real-time ratios relative to the eGFP-transfectants and those for the parental cells was 0.94, indicating their high degree of similarity.

### Transcriptional effects common to wild type and truncated HMGA2

Sixty-six genes were up- and 139 genes were down-regulated by both proteins. A large fraction of the affected genes are known direct targets for NF-κB, and potential intrinsic binding sites for HMGA proteins could be found within many of the NF-κB sites (Additional file 3 Table S3). Most of these genes code for proteins involved in cytokine signalling. All of the genes in the *CXC *chemokine cluster in chromosome band 4q13.3 that were expressed in the parental cell line were down-regulated by both protein variants. Other cytokines that were initially expressed in the parental cells and down-regulated upon HMGA2 expression were *IL1B*, *IL1RN*, *IL6*, *IL11*, *IL15 *and *LIF1*, all containing validated or predicted NF-κB sites and only *IL11 *and *IL15 *lack intrinsic binding sites for HMGA proteins. Several other possible NF-κB targets were down regulated by the over-expression of either form of HMGA2, e.g. *THBS2*, *S100A4*, *IFI44L*, *SOD2*, *PSMB9*, *IGFBP1*, *SERPINB1*, *NFKBIA, BDKRB1 *and *PTGES*, all associated with increased NF-κB activity (http://people.bu.edu/gilmore/nf-kb/target/index.html and references therein). We also observed a four-fold decrease of the primary microRNA miR-146A (MIRN146A), which is known to be regulated by NF-κB [45]. Noteworthy, a less stringent criterion (>2-fold) showed down-regulation of several other potential NF-κB target genes, such as *BCL2A1*, *JUNB*, *LGALS3*, *HAS1*, *TWIST1*, *PTGS2, CFLAR *and *BIC *(encoding miR-155). However, *EDN1, MDK *and *TGM2*, three genes thought to be positively regulated by NF-κB [46-48], were strongly up-regulated in both cell lines.

In addition, several putative interferon-responsive genes were down-regulated, such as *IFI27*, *IFITM1*, *ISGF3G, AIM2, KYNU, C1S *and *OASL*. Since the regulatory region of both *IFI27 *and *IFITM1 *contain IFN-*α *stimulated response element (ISRE; Additional file 4 Table S4) that are recognized by the ISGF3 complex [49] they work downstream of ISGF3G and are therefore strongly dependent on the expression of the *ISGF3G *gene. The *ISGF3G *gene itself is known to be regulated by IL-6 [50] which indicates that these genes are secondary targets to HMGA2.

Another large group of genes that showed expression change was the Krüppel-type zinc finger proteins located in several gene clusters mainly on the long arm of chromosome 19 [51,52]. Some of these zinc finger proteins contain a potent repressor domain, the Krüppel-associated box (KRAB), and they are likely involved in gene silencing [53]. Interestingly, there was a broader up-regulation of several other genes mapped to the chromosome band 19q13 in the cell line over-expressing the truncated HMGA2, including 35 Krüppel-type zinc finger genes of which 27 contain a KRAB domain. This might indicate a common regulatory mechanism for these genes that could involve modulation of chromatin since there was also an increased expression of several unrelated genes near these clusters. Cells expressing HMGA2_WT _had an increased expression of 12 Krüppel-type zinc finger genes, nine of which mapped to the long arm of chromosome 19 and were up-regulated also in HMGA2_TRUNC _transfectants. Six of the common up-regulated genes that mapped to 19q contain a KRAB repressor domain, while the three genes mapped to other chromosome regions, showing elevated expression only in the cells expressing HMGA2_WT_, all contain KRAB domains.

A gene ontology (GO) analysis revealed a highly significant decrease in expression of genes encoding proteins involved in signal transduction (p < 0.001) and response to wounding (p < 0.001), mainly affecting genes involved in cytokine-receptor signalling (p < 0.001), chemotaxis (p < 0.001) and inflammatory response (p < 0.001). In addition, down-regulation of genes involved in regulation of cellular proliferation (p = 0.0033) and epithelial cell differentiation (p = 0.026) was observed. A few genes involved in vascular development (p = 0.033) were up-regulated. However, six out of 66 up-regulated genes encode Krüppel-type zinc finger proteins containing a KRAB repressor domain (p = 0.0051), indicating a common regulatory mechanism for these genes (Table 2).

**Table 2:** Functional annotation analysis of differentially expressed genes

Biological processes affected by over-expression of wild type and truncated HMGA2	
Vascular development (4 of 66 up-regulated genes)	p = 0.039

Response to wounding (17 of 139 down-regulated genes)	p < 0.001
Signal transduction (40/139)	p < 0.001
Chemotaxis (11/139)	p < 0.001
Cytosolic calcium ion homeostasis (5/139)	p < 0.001
Phosphate transport (5/139)	p = 0.0017
Regulation of cell proliferation (11/139)	p = 0.0033
Cell migration (8/139)	p = 0.0057
Cell recognition (3/139)	p = 0.027
Epithelial cell differentiation (3/139)	p = 0.026
Wound healing (4/139)	p = 0.042

**Biological processes affected by over-expression of wild type HMGA2**	

Multi-cellular organismal development (20 of 84 up-regulated genes)	p = 0.0011
Response to hormone stimulus (4/84)	p = 0.0034
Regulation of signal transduction (7/84)	p = 0.022

Pyridine nucleotide biosynthetic process (3 of 99 down-regulated genes)	p = 0.0017
Steroid hormone receptor activity (3/99)	p = 0.0032
Negative regulation of transcription factor activity (3/99)	p = 0.0034
Positive regulation of transcription (7/99)	p = 0.0065
Response to external stimulus (8/99)	p = 0.042

**Biological processes affected by over-expression of truncated HMGA2**	

Regulation of transcription (61 of 258 up-regulated genes)	p < 0.001
Intracellular protein transport across a membrane (6/258)	p = 0.0032
Membrane organization and biogenesis (11/258)	p = 0.011
Ras protein signaling transduction (9/258)	p = 0.031
Regulation of cytoskeleton organization and biogenesis (4/258)	p = 0.042

Antigen processing and presentation (10 of 239 down-regulated genes)	p < 0.001
Multi-cellular organismal development (41/239)	p = 0.0021
Cell adhesion (16/239)	p = 0.019
Eye morphogenesis (3/239)	p = 0.035

The genes were classified into biological processes using gene ontology terms. Each biological process was determined if it was significantly over- or underrepresented (*p *< 0.05) by comparing the observed frequency of genes to the expected frequency of genes on the Affymetrix GeneChip that cover 50 547 known genes. Significantly over- or under-represented processes were grouped and ranked according to the geometric mean of their p-values. A representative annotation term for each cluster, the number of genes associated with the term and its p-value was listed.

### Reciprocal effects of different forms of HMGA2

There were several genes up-regulated by HMGA2_WT _and down- regulated in cells expressing the truncated form, such as *FGF13*, *EHF*, *HCLS1*, *MEST*, *G0S2 *and *PTPRN2*. Two of the gene products, G0S2 and MEST, are both implicated in adipogenesis [54-56] while *FGF13*, *EHF*, *HCLS1 *and *PTPRN2 *are potential regulators of stem cell activity [57] , senescence [58], adhesion [59] and insulin secretion [60], respectively.

### Transcriptional effects specific to full-length HMGA2

Eighty-four genes were up-regulated and 99 genes were down-regulated more than three-fold by wild-type but not truncated HMGA2. Most genes were classified into groups of more general GO terms providing limited information about the underlying processes affected by full-length HMGA2. However, a significant number of the up-regulated genes are cell-type-specific genes involved in embryonal development and likely reflect a transition in the differentiation state of the cells. Some of the down-regulated genes affect gene transcription. However, most of the affected genes were poorly annotated and gave little information about their cellular function.

### Transcriptional effects specific to truncated HMGA2

Two hundred and fifty-eight genes were up-regulated and 239 genes were down-regulated in the cells expressing the truncated protein. HMGA2_TRUNC _cells showed a clear increase in expression of *CXCL12 *whereas the large MHC class II gene cluster was down-regulated (Table 1b). The down-regulation of the MHC class II gene HLA-DRA was verified by qPCR (Table 1b) and using flow cytometry, we could show that HLA-DRA was expressed at a basal level on the surface of both the parental cells and the cells over-expressing the wild type HMGA2, but was strongly down-regulated in HMGA2_TRUNC _cells in according with the expression data (Fig. 1f). Adding interferon *γ*increased the expression of HLA-DRA, resulting in a 40-fold increase in the level on the HMGA2_TRUNC _cells, to give a level of surface protein similar to that on the parental line (not shown).

Based on the microarray data, the SSX gene family appeared to be co-induced in cells expressing HMGA2_TRUNC_, but real-time PCR was only able to verify the induction of SSX1 transcripts. Interesting, the expression increased by 1000-fold indicating a specific activation of this gene.

The GO analysis revealed a significant down-regulation of genes involved in antigen presentation and processing (p < 0.001), and organism development (p = 0.0021). A large proportion of the up-regulated genes encode proteins with roles in regulation of transcription (p < 0.001) and most of them were KRAB-zinc finger proteins. In addition, genes encoding proteins involved in intracellular protein transport across membranes (p = 0.0032), organization of cytoskeletal structures (p = 0.042) and Ras signalling (p = 0.031), were significant up-regulated.

## Discussion

The deregulation of expression caused by the absence of the 3'-UTR in our transgenes, and thus loss of *let-7-*-mediated inhibition, resulted in abundant expression of HMGA2 protein (Fig. 1c). The eGFP fusion proteins were shown to localise in the nucleus just like the endogenous protein. Their distribution to the nucleoli (Fig. 1a, arrowheads) was unexpected though HMGA2 has previously been found to interact with the mainly nucleolar nucleophosmin/B23 protein [61]. Furthermore, the localization of endogenous HMGA2 in the nucleoli was clearly restricted to numerous discrete foci (Fig. 1a, arrow). The distribution of these sites was occasionally scattered throughout the nucleolus or more often restricted to the periphery of the nucleolar body were they seemingly co-localized with B23. In this outer part of nucleolus the late processing of rRNAs takes place before they assemble into ribosomal subunits [62], suggesting that HMGA2 might be involved in these processes. However, this could also be a result of B23 acting as a molecular chaperone [63], sequestering HMGA2 to the nucleolus, as described for IRF-1 [64], pRb [65] and TRF2 [66].

Both wild type and truncated HMGA2 abrogated growth inhibition and adipogenesis (Fig. 1d), probably by a common mechanism. It appears that both HMGA2 proteins prevented the growth arrest necessary for adipogenic differentiation, as cellular protein accumulated at the same rate as in un-induced cells. Interestingly, a recent study showed that the level of HMGA2 is highly regulated during adipogenesis by *let-7 *and knockdown of HMGA2 by this miRNA inhibited both clonal expansion and the transition to terminal differentiation [67]. HMGA2 seems to act in a narrow window early in the adipogenesis where it is involved in expanding the population of preadipocytes and keeps the cells in an undifferentiated state. This would be consistent with our *HMGA2 *over-expressing keeping the mesenchymal stem-like cells in a proliferative state, preventing differentiation into adipocytes. Transgenic mice overexpressing truncated HMGA2 gene, on the other hand, develop well differentiated lipomas and abundant adipose tissue [24]. This discrepancy is not surprising, as the conditions during culture *in vitro *and development *in vivo *are very different, among other things due to the micro¬environment. Furthermore, the absence in hMSC- TERT20 cells of p16, encoded by the *INK4A/ARF *locus, and possibly other oncogenic alterations in our model system [37] may be expected to contribute to the results, although this blockage of differentiation was confirmed in preliminary experiments using another, non-tumourigenic telomerase-immortalised MSC line (Stabell et al, unpublished).

It has recently been reported that HMGA2 represses transcription of *INK4A/ARF *[68] and suggested that this might be mediated through JUNB, a transactivator of *INK4A/ARF *[69]. Consistent with previous findings [3,68], both HMGA2 forms down-regulated the expression of NF-κB regulated genes in the cells, and in addition to binding sites for NF-κB, many of the down-regulated genes identified here, including *JUNB*, also contain potential intrinsic binding sites for HMGA2 (Additional file 3 Table S3). These findings suggest that HMGA2 may repress JUNB by interfering with the NF-κB mediated transactivation of this gene. However, since the *INK4A/ARF *locus is deleted in our cell model, we were not able to confirm an effect on this gene by over-expressing HMGA2. One might speculate that the higher number of genes affected by the truncated protein could be due to a regulatory or moderating function of the c-terminal domain, but this remains to be investigated further.

In spite of abnormal dynamics of growth and differentiation of our model system, we expect the transcription response to HMGA2 overexpression to reflect authentic regulatory functions in an immature mesenchymal context, as these cells show differentiation properties similar to primary mesenchymal stem-like cell cultures.

HMGA2 is known to participate in the formation of heterochromatin in senescent cells [18] and we found that both full-length and truncated HMGA2 proteins co-localized extensively with DAPI-dense regions, probably enriched in heterochromatin (Fig. 1a). The modulation of chromatin by HMGA2 could also be responsible for the coordinated induction of the chromosome 19 KRAB-ZNF genes. Several members of this family modulate cell growth and survival, and they are implicated in malignant disorders [70], although the functions of most of them are largely unknown. Noteworthy, a recently study showed that Apak, encoded by the *ZNF420 *gene, repress p53-mediated apoptosis [71] and it was suggested that various KRAB-type zinc-finger proteins may modulate p53 activity. Although the level of *ZNF420 *transcript increased more than two-fold in cells over-expressing HMGA2, no effects on pro-apoptotic genes were observed (data not shown).

Interestingly, our most up-regulated gene, *SSX1*, encodes a protein that also is involved in chromatin modulation, and is already involved in sarcoma oncogenesis [72]. Members of this gene family are seen expressed in germ cells and mesenchymal stem cells, decrease upon differentiation into adipocytes and osteocytes [73,74], and are expressed in various tumor types [75]. SSX proteins co-localize with Polycomb group (PcG) proteins in the nucleus and likely act through these chromatin regulators to maintain repression of target genes [76,77]. This could suggest that they play a key role in maintaining mesenchymal cell stemness [78], and that increased SSX1 expression could reflect a cancer stem cell phenotype. Although the SSX gene family is highly conserved, the variable activation of SSX genes reported in malignant cells [74] suggests gene-specific transcription control. Their restricted expression pattern is mainly governed by epigenetic silencing [79,80], which could be affected by HMGA2_TRUNC_. An AT-rich region 1 kb upstream from transcription start of SSX1 contains a likely binding site for HMGA2 (^-1076 ^T**ATT**A**A**TAT), although it remains to be proven that it binds specifically to HMGA2_TRUNC_. Recent findings suggest that both HMGA2 [81] and SSX proteins [73,82] are taking part in the epithelial mesenchymal transition, where epithelial cells convert to motile mesenchymal cells [83]. However, the significance of these processes in already mesenchymal cells is unclear. Interestingly, we observed a change in both cell lines to a more restricted but still mesenchymal phenotype by the repression of some epithelial markers, such as keratin 14 (*KRT14*) and the P selectin ligand CD24, and up-regulation of mesenchymal-specific genes such as *CD44*, probably shifting from a multi-lineage potential [84] to a more restricted but proliferative state. The down-regulation of the NF-κB pathway might signify an undifferentiated mesenchymal phenotype committed to adipogenic lineage depending on further signalling cues.

The induction of *CXCL12 *gene expression by HMGA2_TRUNC _is probably the cause of down-regulation of HLA-DRA and other major histocompatibility complex class II (MHC-II) proteins on the cell surface, due to this cytokine's ability to repress the class II transactivator [85]. The fact that interferon induced HLA-DRA protein levels to a similar level in parental and HMGA2_TRUNC _cells supports this interpretation. Loss of MHC-II expression in lymphomas correlates with poor survival [86], and absence of HLA-DR expression is seen in other cancers [87]. Their lost ability to present tumor antigens on their cell surfaces may contribute to their escape from immunosurveillance and therefore this might also provide a selective advantage to sarcoma cells expressing truncated HMGA2.

Since HMGA2 truncation and amplification is observed initially in the development of well-differentiated liposarcomas, borderline tumours of adipocytic differentiation, and always in combination with amplification of the p53-blocking MDM2, we do not expect a very "malignant" phenotype when HMGA2 is overexpressed on its own. On the other hand, a phenotype where adipogenesis is inhibited would be consistent with a role in adipogenic tumours. When truncated HMGA2 is overexpressed in transgenic mice, they develop adipose hypertrophy, which indicates that adipocytic differentiation is not completely blocked [24]. Like our constructs, theirs lack the 3' UTR, thus preventing down regulation of HMGA2 by *let-7 *as differentiation progresses, and the most likely explanation for the discrepancy is that the balance of proliferation and differentiation over weeks or months *in vivo *is sufficiently different for our short-term *in vitro *conditions, or there are differences in how human and mouse cells are regulated at this level. Although we get the same result in another non-transformed hMSC line, this model is also based on ectopic activation of telomerase, which could affect the result[88,89].

To investigate these questions, we will determine the activity of truncated HMGA2 in sarcoma cells by siRNA-based knock-down in cells with the natural malignant genetic background.

## Conclusions

Our results show that over-expression of truncated HMGA2 induces a more mesenchymal (stem-like) phenotype, characterized by resistance toward differentiation, over-expression of SSX1, lost expression of certain epithelial markers and strengthened expression of mesenchymal markers. We suggest that amplification and truncation of HMGA2 in sarcoma provide the tumors with a more stem-like phenotype.

## Competing interests

The authors declare that they have no competing interests.

## Authors' contributions

JH participated in the planning of the experiments, performed all the transfections and experiments for array analysis, performed most of the array data analysis, and writing of the manuscript. MS performed the differentiation analysis and participated in the writing of the manuscript. LAMZ conceived the study, supervised the practical work, and participated in writing of the manuscript. SAUL performed the confocal microscopy analyses and participated in the writing of the manuscript. MK constructed and provided the mesenchymal model system used, and critically read the manuscript. OM conceived the study, supervised the practical work, and participated in the writing of the manuscript. All authors read and approved the final manuscript.

## Pre-publication history

The pre-publication history for this paper can be accessed here:

http://www.biomedcentral.com/1471-2407/10/329/prepub

## Supplementary Material

Additional file 1 **Additional Table S1 TaqMan primers for real-time PCR**. Click here for file

Additional file 2 **Additional Table S2 The complete list of transcripts changed more than 3-fold**. Click here for file

Additional file 3 **Additional Table S3 Predicted NF-κB recognition sequences in affected genes**. Click here for file

Additional file 4 **Additional Table S4 Predicted STAT2 recognitions sequences in affected genes**. Click here for file
