# Supplementary material for: Identification of target genes for wild type and truncated HMGA2 in mesenchymal stem-like cells
Source: BMC Cancer. 2010 Jun 25;10:329. doi: 10.1186/1471-2407-10-329 (PMC2912264; doi:10.1186/1471-2407-10-329)
Supplement: Additional file 1 — Additional Table S1 TaqMan primers for real-time PCR. [file 1471-2407-10-329-S1.DOC]

**Supplementary table 1 TaqMan primers for real-time PCR**

*Gene name* TaqMan assay Location Log_2_-fold change (ΔΔC_T_)

F T

*ZBED2* Hs00976682_s1 exon 2 4.5 1.3

*LCP1* Hs00158701_m1 exon 10-11 3.9 -5.8

*MDK* Hs00171064_m1 exon 4-5 1.5 2.6

*HDAC9* Hs01081558_m1 exon 9-10 1.5 0.9

*G0S2* Hs00377852_g1 exon 1-2 2.9 -6.2

*CDK6* Hs01026373_m1 exon 6-7 0.9 1.4

*FGF1* Hs00265254_m1 exon 3-4 0.6 1.5

*FGF1* Hs00361127_m1 exon 2-4 0.8 1.5

*SSX3* Hs01934296_s1 exon 8 0 0.5

*SSX2* Hs00817683_m1 exon 5-6 ND ND^1^

*FGF13* Hs00182807_m1 exon 3-4 4.6 0.5

*ASXL1* Hs00898213_m1 exon 10-11 0.3 0.4

*HMGA2* Hs00171569_m1 exon 2-3 0.7 0.7

*HMGA2* Hs00971725_m1 exon 4-5 0.9 0

*RAP1A* Hs01092205_g1 exon 6-7 0 0

*GAPDH* 4326317E 0 0

*CCNA2* Hs00153138_m1 exon 1-2 0 0

*ERCC1* Hs00157415_m1 exon 9-10 0 0.4

*IGF2BP2* Hs00538956_m1 exon 10-11 -0.3 0.3

*PKIB* Hs00261162_m1 exon 6-7 -1.7 -4.9

*B2M* 4326319E -0.7 -0.9

*DLX2* Hs00269993_m1 exon 1-2 -0.5 0.6

*SSX1* Hs00846692_s1 exon 4 0.9 9.6

*IL8* Hs00174103_m1 exon 1-2 -7.1 -4.0

*HLA-DRA* Hs00219575_m1 exon 1-2 -0.4 -8.1

*PBX1* Hs00231228_m1 exon 4-5 -1.6 -2.0

*FGF1* Hs00361126_m1 exon 1-2 0.4 -2.6

*JUNB* Hs00357891_s1 exon 1 -2.0 -1.4

*BAI3* Hs00938878_m1 exon 11-12 4.4 4.6

*Gene name* TaqMan assay Location Log_2_-fold change (ΔΔC_T_)

F T

*CD74* Hs00269961_m1 exon 1-2 -0.3 -5.4

*KLF9* Hs00230918_m1 exon 1-2 -1.8 -2.0

*HLA-DPA1* Hs00410276_m1 exon 4-5 -0.4 -6.8

*IL1RN* Hs00277299_m1 exon 3-4 -4.4 -3.2

*VAV3* Hs00196125_m1 exon 20-21 -5.8 -7.2

*SSX4* Hs02341529_g1 exon 2-3 -2.0 -1.1

*MEST* Hs00853380_g1 exon 12 2.4 -3.1

*SMAD6* Hs00178579_m1 exon 2-3 -2.6 -2.9

*KIAA1644* Hs00286734_m1 exon 3-4 -3.4 -3.1

*IL1B* Hs00174097_m1 exon 5-6 -2.5 -4.3

*BMP6* Hs00233470_m1 exon 2-3 -4.2 -4.1

*CXCL6* Hs00237017_m1 exon 3-4 -6.7 -6.3

*GATA3* Hs00231122_m1 exon 2-3 3.6 -1.6

*CXCL1* Hs00605382_gH exon 3-4 ND -4.8

*CXCL12* Hs00171022_m1 exon 2-3 -1.0 2.7

^1^ND= not determined due to no transcripts detected by the TaqMan assay
