# Supplementary material for: Identification of target genes for wild type and truncated HMGA2 in mesenchymal stem-like cells
Source: BMC Cancer. 2010 Jun 25;10:329. doi: 10.1186/1471-2407-10-329 (PMC2912264; doi:10.1186/1471-2407-10-329)
Supplement: Additional file 2 — Additional Table S2 The complete list of transcripts changed more than 3-fold. [file 1471-2407-10-329-S2.PDF]

Up-regulated genes in F vs P

Probe set	Gene	Gene ID	Public ID	Fold change (log2 ratio)
1555340_x_at	GFP	n/a	AB05184(	6,74
1555339_at	GFP	n/a	AB05184(	6,41
219836_at	ZBED2	79413	NM_0245	5,33
204967_at	SHROOM2	357	NM_0016	4,63
205110_s_at	FGF13	2258	NM_0041	4,50
205638_at	BAI3	577	NM_0017	4,47
228104_at	PLXNA4A	57671	AL117427	4,41
208885_at	LCP1	3936	J02923	4,11
1553311_at	C20orf197	284756	NM_1736	4,01
213524_s_at	G0S2	50486	NM_0157	3,72
205659_at	HDAC9	9734	NM_0147	3,68
215856_at	SIGLEC15	284266	AK02583:	3,65
202957_at	HCLS1	3059	NM_0053	3,59
1559462_at	LOC6463	646340	BC04341:	3,56
209604_s_at	GATA3	2625	BC00307(	3,54
225354_s_at	SH3BGRL	83699	AL03570C	3,49
203708_at	PDE4B	5142	NM_0026	3,45
232027_at	SYNE1	23345	AL049548	3,23
226219_at	ARHGAP3	257106	AW57512	3,19
207149_at	CDH12	1010	L33477	3,18
228051_at	LOC2024!	202451	AI979261	3,07
235004_at	RBM24	221662	AI677701	3,07
237737_at	LOC3750:	375010 /,	AI359676	3,03
1552721_a_at	FGF1	2246	NM_0331	3,03
1564838_a_at	LOC1517(	151760	BC03857:	3,00
202668_at	EFNB2	1948	BF00167C	2,93
219315_s_at	C16orf30	79652	NM_0246	2,93
203030_s_at	PTPRN2	5799	AF00755!	2,90
203288_at	KIAA0355	9710	NM_0146	2,88
202669_s_at	EFNB2	1948	U16797	2,85
218995_s_at	EDN1	1906	NM_0019	2,85
242705_x_at	n/a	n/a	AI188104	2,78
211003_x_at	TGM2	7052	BC00355:	2,77
232360_at	EHF	26298	AA56514:	2,76
222802_at	EDN1	1906	J05008	2,74
228083_at	CACNA2D	93589	AI433691	2,74
219369_s_at	OTUB2	78990	NM_0231	2,73
232361_s_at	EHF	26298	AA56514:	2,67
32699_s_at	PVR	5817	X64116	2,66
231325_at	UNC5D	137970	AI802048	2,61
219308_s_at	AK5	26289	NM_0120	2,60
212238_at	ASXL1	171023	AL117518	2,59
1552912_a_at	IL23R	149233	NM_1447	2,55
232014_at	ZNF30	90075	AI700188	2,53
222227_at	FLJ20840	n/a	AK00084:	2,51
202016_at	MEST	4232	NM_0024	2,50
232315_at	LOC4007:	400713	AU14971:	2,45
214009_at	MSL3L1	10943	R10150	2,44
201042_at	TGM2	7052	AL031651	2,43
217705_at	PRKD1	5587	AW08517	2,42
237552_at	n/a	n/a	BF05647:	2,41
239135_at	n/a	n/a	AI675054	2,37
243529_at	MARS2	92935	BE542381	2,34
207038_at	SLC16A6	9120	NM_0046	2,33

1568873_at	ZNF519	162655	BC01070!	2,33
220230_s_at	CYB5R2	51700	NM_0162	2,33
222686_s_at	FLJ11151	55313	AL572407	2,31
205547_s_at	TAGLN	6876	NM_0031	2,30
226028_at	ROBO4	54538	AA15602	2,30
205832_at	CPA4	51200	NM_0163	2,30
227915_at	ASB2	51676	AI872284	2,30
237290_at	n/a	n/a	AW13887	2,29
229429_x_at	FAM91A2	57234	AA86322	2,29
212092_at	PEG10	23089	BE85818	2,28
217223_s_at	BCR	613	U07000	2,28
234685_x_at	KRTAP4-9	85286	AJ406941	2,28
212599_at	AUTS2	26053	AK02529	2,27
203029_s_at	PTPRN2	5799	NM_0028	2,26
37028_at	PPP1R15A	23645	U83981	2,26
1554757_a_at	INPP5A	3632	AF27305	2,26
1555154_a_at	QKI	9444	AF142421	2,25
209031_at	CADM1	23705	AL51971	2,24
231098_at	n/a	n/a	BF93999	2,22
230748_at	SLC16A6	9120	AI873273	2,22
231879_at	COL12A1	1303	AL096771	2,19
1557756_a_at	C14orf14	145508	AW02833	2,19
219014_at	PLAC8	51316	NM_0166	2,17
221667_s_at	HSPB8	26353	AF133207	2,17
222450_at	TMEPAI	56937	AL035541	2,17
1554433_a_at	ZNF146	7705	BC00515	2,16
204433_s_at	SPATA2	9825	U28164	2,15
211573_x_at	TGM2	7052	M98478	2,15
212636_at	QKI	9444	AL031781	2,14
1557261_at	WHDC1L1	339005 /,	AK09125	2,14
222449_at	TMEPAI	56937	AL035541	2,13
235086_at	THBS1	7057	AW95658	2,12
225645_at	EHF	26298	AI763378	2,12
1561853_a_at	IL23R	149233	BC01682	2,12
1555007_s_at	WDR66	144406	BC03623	2,11
227920_at	KIAA1553	57673	AV70088	2,10
241418_at	LOC34488	344887	AI819386	2,09
1555724_s_at	TAGLN	6876	BC01094	2,09
201537_s_at	DUSP3	1845	BC00268	2,07
231856_at	KIAA1244	57221	AB03307	2,07
214612_x_at	MAGEA6	4105	U10691	2,06
227670_at	ZNF75A	7627	N74222	2,06
202923_s_at	GCLC	2729	NM_0014	2,05
219495_s_at	ZNF180	7733	NM_0132	2,04
206155_at	ABCC2	1244	NM_0003	2,04
243195_s_at	ZNF551	90233	BF438407	2,02
201538_s_at	DUSP3	1845	NM_0040	2,01
241871_at	CAMK4	814	AL52910	1,97
236489_at	n/a	n/a	AI282097	1,97
216278_at	n/a	n/a	AL109705	1,95
1554195_a_at	MGC2398	389336	BC02168	1,95
239201_at	ALS2CR7	65061	AI028242	1,94
211534_x_at	PTPRN2	5799	U65065	1,94
1559433_at	LOC1497	149773	AK09170	1,94
201107_s_at	THBS1	7057	AI812030	1,93
231950_at	ZNF658	26149	AW87463	1,93

226876_at	FAM101B	359845	AI961778	1,91
227174_at	WDR72	256764	Z98443	1,90
214940_s_at	SMG6	23293	AB01827[illegible]	1,90
205523_at	HAPLN1	1404	U43328	1,90
217289_s_at	SLC37A4	2542	AF097831	1,89
223630_at	C7orf13	129790	AF06359[illegible]	1,89
238944_at	n/a	n/a	AI393706	1,89
221014_s_at	RAB33B	83452	NM_0312	1,88
204875_s_at	GMDS	2762	NM_0015	1,88
211681_s_at	PDLIM5	10611	AF11670[illegible]	1,87
235484_at	PTAR1	375743	BE89288[illegible]	1,86
211975_at	ZNF289	84364	BE29967[illegible]	1,85
235063_at	C20orf19[illegible]	149840	AA85672[illegible]	1,85
207131_x_at	GGT1	2678	NM_0134	1,85
225055_at	n/a	n/a	AV73524[illegible]	1,85
232434_at	DIRC3	729582	AA16094[illegible]	1,85
209185_s_at	IRS2	8660	AF07331[illegible]	1,85
230773_at	n/a	n/a	AA62851[illegible]	1,85
244462_at	ZNF224	7767	AA81198[illegible]	1,83
239336_at	THBS1	7057	BF10973[illegible]	1,82
224848_at	CDK6	1021	AA92206[illegible]	1,82
206371_at	FOLR3	2352	NM_0008	1,82
208879_x_at	PRPF6	24148	BG46903[illegible]	1,82
202672_s_at	ATF3	467	NM_0016	1,81
233501_at	n/a	n/a	AL13747[illegible]	1,81
205508_at	SCN1B	6324	NM_0010	1,81
206421_s_at	SERPINB[illegible]	8710	NM_0037	1,80
227196_at	RHPN2	85415	BG05498[illegible]	1,80
203123_s_at	SLC11A2	4891	AU15446[illegible]	1,80
214443_at	PVR	5817	NM_0065	1,80
202083_s_at	SEC14L1	6397	AI017770	1,80
208284_x_at	GGT1	2678	NM_0134	1,80
205264_at	CD3EAP	10849	NM_0120	1,79
1554287_at	TRIM4	89122	BC01176[illegible]	1,79
244664_at	n/a	n/a	AA41228[illegible]	1,79
235648_at	ZNF567	163081	AA74265[illegible]	1,79
229744_at	SSFA2	6744	AL556611	1,78
230508_at	DKK3	27122	AL569601	1,78
242375_x_at	n/a	n/a	AI796189	1,78
226991_at	NFATC2	4773	AA48968[illegible]	1,78
209706_at	NKX3-1	4824	AF24770[illegible]	1,77
229845_at	MAPKAP1	79109	BF93991[illegible]	1,76
213805_at	ABHD5	51099	AI692428	1,76
209314_s_at	HBS1L	10767	AK02425[illegible]	1,76
209030_s_at	CADM1	23705	NM_0143	1,76
211959_at	IGFBP5	3488	AW00753	1,75
204614_at	SERPINB[illegible]	5055	NM_0025	1,75
225647_s_at	CTSC	1075	AI246687	1,75
225564_at	SPATA13	221178	AW26939	1,75
212813_at	JAM3	83700	AA14964[illegible]	1,75
225296_at	ZNF317	57693	AB04680[illegible]	1,74
212133_at	NIPA2	81614	AI681536	1,74
230123_at	NECAP2	55707	AI608836	1,74
203895_at	PLCB4	5332	AL53511[illegible]	1,74
244640_at	LOC3428[illegible]	342892	AW44039	1,73
1558700_s_at	ZNF260	339324	BC04267[illegible]	1,73

203221_at	TLE1	7088	AI758763	1,73

Down-regulated genes in F vs P

Probe set	Gene	Gene ID	Public ID	Fold change (log2 ratio)
206336_at	CXCL6	6372	NM_0029	-8,44
202859_x_at	IL8	3576	NM_0005	-7,54
209771_x_at	CD24	934	AA76118	-7,07
211506_s_at	IL8	3576	AF04333	-6,59
227058_at	C13orf33	84935	AW08473	-6,46
210367_s_at	PTGES	9536	AF01031	-6,33
219049_at	ChGn	55790	NM_0183	-5,55
205207_at	IL6	3569	NM_0006	-5,48
216379_x_at	CD24	934	AK00016	-5,44
204491_at	PDE4D	5144	R40917	-5,18
212657_s_at	IL1RN	3557	U65590	-5,14
209616_s_at	CES1	1066	S73751	-5,06
221901_at	KIAA164	85352	BF51607	-4,97
220330_s_at	SAMSN1	64092	NM_0221	-4,79
205576_at	SERPIND	3053	NM_0001	-4,67
52837_at	KIAA164	85352	AL04702	-4,62
217562_at	FAM5C	339479	BF58952	-4,38
224989_at	n/a	n/a	AI824013	-4,34
228501_at	GALNTL2	117248	BF05534	-4,30
205081_at	CRIP1	1396	NM_0013	-4,26
208650_s_at	CD24	934	BG32786	-4,14
227404_s_at	EGR1	1958	AI459194	-4,06
266_s_at	CD24	934	L33930	-3,96
209409_at	GRB10	2887	D86962	-3,93
209774_x_at	CXCL2	2920	M57731	-3,88
238332_at	ANKRD29	147463	AI307802	-3,83
1555778_a_at	POSTN	10631	AY14064	-3,82
206513_at	AIM2	9447	NM_0048	-3,76
207316_at	HAS1	3036	NM_0015	-3,74
230109_at	PDE7B	27115	AI638433	-3,72
207510_at	BDKRB1	623	NM_0007	-3,71
210809_s_at	POSTN	10631	D13665	-3,70
207850_at	CXCL3	2921	NM_0020	-3,58
205234_at	SLC16A4	9122	NM_0046	-3,57
218820_at	C14orf13	56967	NM_0202	-3,57
209351_at	KRT14	3861	BC00269	-3,48
223179_at	YPEL3	83719	BC00500	-3,41
229414_at	PITPNC1	26207	AI676095	-3,41
236361_at	GALNTL2	117248	BF43237	-3,40
203108_at	GPRC5A	9052	NM_0039	-3,37
227238_at	MUC15	143662	W93847	-3,37
226682_at	LOC2836	283666	AW00618	-3,35
229308_at	n/a	n/a	AW27379	-3,33
1554067_at	FLJ32549	144577	BC03624	-3,30
203083_at	THBS2	7058	NM_0032	-3,29
205302_at	IGFBP1	3484	NM_0005	-3,28
209277_at	TFPI2	7980	AL57409	-3,23
224990_at	C4orf34	201895	BE97272	-3,21
227059_at	GPC6	10082	AI651255	-3,19
230383_x_at	n/a	n/a	AA13328	-3,18
213488_at	SNED1	25992	N73970	-3,17
227052_at	n/a	n/a	AI810669	-3,16
219799_s_at	DHRS9	10170	NM_0057	-3,15
232748_at	PAPPA	5069	AU15672	-3,13

209755_at	NMNAT2	23057	AF288395	-3,13
221748_s_at	TNS1	7145	AL046979	-3,11
205992_s_at	IL15	3600	NM_0005	-3,09
209335_at	DCN	1634	AI281593	-3,08
218162_at	OLFML3	56944	NM_0201	-3,07
230054_at	PRRT1	80863	AW13449	-3,04
208651_x_at	CD24	934	M58664	-3,03
218807_at	VAV3	10451	NM_0061	-3,02
215462_at	PLK3	1263	AI978990	-3,02
208747_s_at	C1S	716	M18767	-3,01
223952_x_at	DHRS9	10170	AF240698	-2,96
223093_at	ANKH	56172	T99215	-2,95
209278_s_at	TFPI2	7980	L27624	-2,94
212148_at	PBX1	5087	AL049381	-2,94
221019_s_at	COLEC12	81035	NM_0307	-2,92
1556935_at	n/a	n/a	AF085839	-2,88
230799_at	LOC15083	150837	W72564	-2,87
227074_at	n/a	n/a	AA524669	-2,87
235227_at	n/a	n/a	AI025829	-2,86
203543_s_at	KLF9	687	NM_0012	-2,80
206785_s_at	KLRC1; K	3821; 382	NM_0022	-2,78
214078_at	n/a	n/a	AF070581	-2,76
204439_at	IFI44L	10964	NM_0068	-2,76
212314_at	KIAA0746	23231	AB018289	-2,76
231979_at	n/a	n/a	AU155091	-2,73
228155_at	C10orf58	84293	BF512388	-2,73
1556329_a_at	n/a	n/a	BC042378	-2,72
204457_s_at	GAS1	2619	NM_0020	-2,70
206924_at	IL11	3589	NM_0006	-2,70
201744_s_at	LUM	4060	NM_0023	-2,70
212444_at	n/a	n/a	AA156240	-2,69
220076_at	ANKH	56172	NM_0198	-2,69
204463_s_at	EDNRA	1909	AU118882	-2,69
236277_at	n/a	n/a	H23551	-2,68
207761_s_at	METTL7A	25840	NM_0140	-2,67
232504_at	miR-146a	406938	AL389942	-2,66
200962_at	RPL31	6160	AI348010	-2,66
207069_s_at	SMAD6	4091	NM_0055	-2,64
218976_at	DNAJC12	56521	NM_0218	-2,62
39402_at	IL1B	3553	M15330	-2,62
219990_at	E2F8	79733	NM_0246	-2,60
203126_at	IMPA2	3613	NM_0142	-2,60
235476_at	TRIM59	286827	AW18245	-2,57
204470_at	CXCL1	2919	NM_0015	-2,56
203416_at	CD53	963	NM_0005	-2,55
203882_at	ISGF3G	10379	NM_0060	-2,55
209469_at	GPM6A	2823	BF939489	-2,54
238504_at	C6orf57	135154	AA521023	-2,53
213215_at	n/a	n/a	AI910895	-2,53
207388_s_at	PTGES	9536	NM_0048	-2,53
213355_at	ST3GAL6	10402	AI989567	-2,52
206026_s_at	TNFAIP6	7130	NM_0071	-2,51
214920_at	THSD7A	221981	R33964	-2,51
212913_at	C6orf26 /.	401251 /,	BE674960	-2,49
229152_at	C4orf7	260436	AI718421	-2,48
202202_s_at	LAMA4	3910	NM_0022	-2,48

225975_at	PCDH18	54510	AW18988	-2,48
204977_at	DDX10	1662	NM_0043	-2,46
228708_at	RAB27B	5874	BF438386	-2,46
205067_at	IL1B	3553	NM_0005	-2,45
205584_at	CXorf45	79868	NM_0248	-2,45
227062_at	TncRNA	283131	AU15536	-2,44
1555786_s_at	n/a	n/a	BC00803	-2,42
206176_at	BMP6	654	NM_0017	-2,42
209016_s_at	KRT7	3855	BC00270	-2,42
221063_x_at	RNF123	63891	NM_0220	-2,42
213493_at	SNED1	25992	BF509657	-2,42
211161_s_at	COL3A1	1281	AF130082	-2,41
209508_x_at	CFLAR	8837	AF005774	-2,41
228316_at	FLJ31438	130162	AA90547	-2,40
223843_at	SCARA3	51435	AB00783	-2,40
222784_at	SMOC1	64093	AJ249900	-2,38
235740_at	n/a	n/a	BG25058	-2,37
218983_at	C1RL	51279	NM_0165	-2,34
207231_at	DZIP3	9666	NM_0146	-2,33
204464_s_at	EDNRA	1909	NM_0019	-2,33
218764_at	PRKCH	5583	NM_0240	-2,32
202411_at	IFI27	3429	NM_0055	-2,31
214761_at	ZNF423	23090	AW14941	-2,29
209681_at	SLC19A2	10560	AF15333	-2,29
208949_s_at	LGALS3	3958	BC00112	-2,28
223395_at	ABI3BP	25890	AB05610	-2,27
213621_s_at	GUK1	2987	AW18289	-2,27
235274_at	n/a	n/a	AA74063	-2,27
213396_s_at	n/a	n/a	AA45692	-2,27
235889_at	n/a	n/a	AI825987	-2,27
202388_at	RGS2	5997	NM_0029	-2,26
210663_s_at	KYNU	8942	BC00087	-2,26
231120_x_at	PKIB	5570	AL569326	-2,25
204359_at	FLRT2	23768	NM_0132	-2,24
213661_at	DKFZP58	25891	AI671186	-2,24
204035_at	SCG2	7857	NM_0034	-2,23
215076_s_at	COL3A1	1281	AU14416	-2,22
227688_at	LRCH2	57631	AK02212	-2,21
238478_at	BNC2	54796	H97386	-2,21
224314_s_at	EGLN1	54583	AF277174	-2,21
206833_s_at	ACYP2	98	NM_0011	-2,21
204821_at	BTN3A3	10384	NM_0069	-2,19
230866_at	CYSLTR1	10800	BE54954	-2,19
204485_s_at	TOM1L1	10040	NM_0054	-2,19
206027_at	S100A3	6274	NM_0029	-2,19
217388_s_at	KYNU	8942	D55639	-2,19
205119_s_at	FPR1	2357	NM_0020	-2,16
200878_at	EPAS1	2034	AF052094	-2,16
237062_at	n/a	n/a	BE22210	-2,16
244025_at	n/a	n/a	BF590917	-2,15
242652_at	n/a	n/a	AI760942	-2,15
212899_at	CDC2L6	23097	AB02895	-2,15
219023_at	C4orf16	55435	NM_0185	-2,14
206926_s_at	IL11	3589	M57765	-2,13
243438_at	PDE7B	27115	BE96857	-2,13
227067_x_at	NOTCH2N	388677	AW02496	-2,13

221747_at	TNS1	7145	AL046979	-2,12
208394_x_at	ESM1	11082	NM_0070	-2,11
31637_s_at	NR1D1 //,	7067 /// !	X72631	-2,11
226849_at	DENND1A	57706	AB046828	-2,10
1563246_at	n/a	n/a	BC038192	-2,10
233068_at	n/a	n/a	AK023264	-2,10
225600_at	LOC28614	286144	AW30330	-2,10
235956_at	KIAA1377	57562	AI797063	-2,10
228156_at	n/a	n/a	AW34207	-2,09
211742_s_at	EVI2B	2124	BC005926	-2,09
203542_s_at	KLF9	687	AI690205	-2,09
209906_at	C3AR1	719	U62027	-2,08
223204_at	C4orf18	51313	AF260333	-2,08
1566342_at	n/a	n/a	R34841	-2,08
1557478_at	n/a	n/a	BM97713	-2,07
203143_s_at	KIAA0040	9674	T79953	-2,07
1568983_a_at	n/a	n/a	BI547087	-2,06
242239_at	n/a	n/a	AW97088	-2,06
209734_at	NCKAP1L	3071	BC001604	-2,06
223551_at	PKIB	5570	AF225513	-2,06
207147_at	DLX2	1746	NM_0044	-2,06
214022_s_at	IFITM1	8519	AA749101	-2,05
213222_at	PLCB1	23236	AL049593	-2,05
234989_at	TncRNA	283131	AV699657	-2,05
232138_at	MBNL2	10150	AW27691	-2,04
242894_at	n/a	n/a	AA620926	-2,04
225599_s_at	LOC28614	286144	AW30330	-2,04
231001_at	FIBIN	387758	AI755024	-2,03
238902_at	n/a	n/a	T85248	-2,03
205682_x_at	APOM	55937	NM_0191	-2,03
204719_at	ABCA8	10351	NM_0071	-2,02
227593_at	FLJ37453	645580	AW96447	-2,02
237563_s_at	LOC44073	440731	AI286239	-2,01
202273_at	PDGFRB	5159	NM_0026	-2,01
225603_s_at	LOC28614	286144	BE962119	-2,00
229710_at	n/a	n/a	AA843541	-1,99
1562013_a_at	n/a	n/a	AK074451	-1,99
201502_s_at	NFKBIA	4792	AI078167	-1,99
218086_at	NPDC1	56654	NM_0153	-1,98
212151_at	PBX1	5087	BF967998	-1,97
221606_s_at	NSBP1	79366	BC005342	-1,97
204059_s_at	ME1	4199	NM_0023	-1,97
238178_at	n/a	n/a	BF110268	-1,97
1553778_at	WBSCR27	155368	NM_1525	-1,97
225842_at	PHLDA1	22822	AK026181	-1,96
209772_s_at	CD24	934	X69397	-1,96
212343_at	YIPF6	286451	AL117461	-1,96
203919_at	TCEA2	6919	NM_0031	-1,95
1555852_at	n/a	n/a	AI375915	-1,94
1555920_at	CBX3	11335	BU683892	-1,94
221911_at	ETV1	2115	BE881590	-1,94
1555967_at	n/a	n/a	AA362254	-1,93
205909_at	POLE2	5427	NM_0026	-1,92
207826_s_at	ID3	3399	NM_0021	-1,92
230118_at	n/a	n/a	AA669158	-1,92
203412_at	LZTR1	8216	NM_0067	-1,92

223833_at	WDR55	54853	BC002482	-1,92
234411_x_at	CD44	960	U94903	-1,91
230333_at	n/a	n/a	BE326919	-1,90
213496_at	LPPR4	9890	AW59256	-1,90
238520_at	TRERF1	55809	BF724270	-1,90
201893_x_at	DCN	1634	AF138300	-1,89
235428_at	n/a	n/a	H78106	-1,89
228927_at	ZNF397	84307	AW29141	-1,89
224435_at	C10orf58	84293	BC005871	-1,89
211896_s_at	DCN	1634	AF138302	-1,88
230793_at	LRRC16	55604	BE671038	-1,88
210592_s_at	SAT1	6303	M55580	-1,86
238649_at	PITPNC1	26207	AA815089	-1,86
236462_at	n/a	n/a	AA742310	-1,86
223194_s_at	C6orf85	63027	AL512737	-1,86
1559921_at	PECAM1	5175	AW13891	-1,86
229787_s_at	OGT	8473	AI742039	-1,85
202499_s_at	SLC2A3	6515	NM_0069	-1,85
226238_at	MCEE	84693	AI934339	-1,84
233090_at	n/a	n/a	AU144140	-1,83
202887_s_at	DDIT4	54541	NM_0190	-1,83
229201_at	n/a	n/a	AW04465	-1,83
203186_s_at	S100A4	6275	NM_0029	-1,83
212613_at	BTN3A2	11118	AI991252	-1,83
203455_s_at	SAT1	6303	NM_0029	-1,83
226454_at	MARCH-9	92979	BF508604	-1,82
203763_at	DYNC2LI1	51626	NM_0160	-1,82
209894_at	LEPR	3953	U50748	-1,82
213988_s_at	SAT1	6303	BE971383	-1,82
232382_s_at	PCMTD1	115294	BE150929	-1,81
202708_s_at	HIST2H2E	8349	NM_0035	-1,80
211813_x_at	DCN	1634	AF138303	-1,80
231779_at	IRAK2	3656	AI246590	-1,80
238612_at	n/a	n/a	AW29807	-1,80
210794_s_at	MEG3	55384	AF119863	-1,80
204385_at	KYNU	8942	NM_0039	-1,79
239738_at	DACH2	117154	AW78000	-1,79
220301_at	CCDC102	79839	NM_0247	-1,77
230861_at	DKFZP434	26082	AA889613	-1,77
213110_s_at	COL4A5	1287	AW05217	-1,76
206953_s_at	LPHN2	23266	NM_0123	-1,76
57715_at	FAM26B	51063	W72694	-1,76
224794_s_at	CERCAM1	51148	AA654142	-1,76
211329_x_at	HFE	3077	AF115264	-1,75
226210_s_at	MEG3	55384	AI291123	-1,75
209676_at	TFPI	7035	J03225	-1,75
214486_x_at	CFLAR	8837	AF041459	-1,74
203765_at	GCA	25801	NM_0121	-1,74
226636_at	PLD1	5337	AI378587	-1,73
1559725_at	n/a	n/a	AL832797	-1,73
1554145_a_at	CCDC128	129285	BC040721	-1,73
201852_x_at	COL3A1	1281	AI813758	-1,73
212419_at	C10orf56	219654	AA131324	-1,73
1558624_at	n/a	n/a	BC033250	-1,73

Up-regulated genes in T vs P

Probe set	Gene	Gene ID	Public ID	Fold change (log2 ratio)
1555339_at	GFP	n/a	AB05184€	7,08
1555340_x_at	GFP	n/a	AB05184€	7,00
231597_x_at	spacer tra	n/a	AI371550	6,72
210394_x_at	SSX4 ///	548313 /,	BC00532!	6,47
209687_at	CXCL12	6387	U19495	6,37
211425_x_at	SSX4 ///	548313 /,	AF25750(	6,12
207638_at	PRSS7	5651	NM_0027	6,02
207601_at	SULT1B1	27284	NM_0144	5,77
210497_x_at	SSX2	6757	BC00281{	5,74
216471_x_at	SSX2	6757	X79200	5,68
217269_s_at	PRSS7	5651	AP00167z	5,26
206626_x_at	SSX1	6756	BC00100:	4,91
206627_s_at	SSX1	6756	NM_0056	4,76
205828_at	MMP3	4314	NM_0024	4,68
220230_s_at	CYB5R2	51700	NM_0162	4,46
205563_at	KISS1	3814	NM_0022	4,45
222802_at	EDN1	1906	J05008	4,45
232027_at	SYNE1	23345	AL04954{	4,43
205524_s_at	HAPLN1	1404	NM_0018	4,42
1562056_at	n/a	n/a	BU95506:	4,39
205523_at	HAPLN1	1404	U43328	4,32
239046_at	n/a	n/a	AA32224!	4,31
205638_at	BAI3	577	NM_0017	4,23
1559462_at	n/a	n/a	BC04341:	4,08
230204_at	HAPLN1	1404	AU14411‹	4,01
1552721_a_at	FGF1	2246	NM_0331	3,94
239336_at	THBS1	7057	BF10973z	3,94
204388_s_at	MAOA	4128	NM_0002	3,89
213764_s_at	MFAP5	8076	AW66589	3,89
211670_x_at	SSX3	10214	S82471	3,83
240512_x_at	KCTD4	386618	H10766	3,78
204507_s_at	PPP3R1	5534	NM_0009	3,78
244640_at	LOC34289	342892	AW44039	3,74
239250_at	ZNF542	147947	BE96603{	3,72
231738_at	PCDHB7	56129	NM_0189	3,71
1552912_a_at	IL23R	149233	NM_1447	3,69
209758_s_at	MFAP5	8076	U37283	3,68
227850_x_at	CDC42EP!	148170	AW08454	3,65
204220_at	GMFG	9535	NM_0048	3,65
209875_s_at	SPP1	6696	M83248	3,61
218995_s_at	EDN1	1906	NM_0019	3,60
235358_at	LOC7284{	728485	AW96120	3,57
212741_at	MAOA	4128	AA92335‹	3,55
235414_at	ZNF383	163087	BF432571	3,40
231943_at	ZFP28	140612	AA83132:	3,34
237737_at	LOC3750:	375010 /,	AI359676	3,34
212570_at	ENDOD1	23052	AL573201	3,33
244664_at	n/a	n/a	AA41228:	3,33
232774_x_at	ZIK1	284307	AC00368z	3,30
238751_at	n/a	n/a	AI343000	3,25
209949_at	NCF2	4688	BC00160€	3,25
207528_s_at	SLC7A11	23657	NM_0143	3,23
234937_x_at	ZFP28	140612	AC00722{	3,22
232014_at	ZNF30	90075	AI700188	3,19

239135_at	---	---	AI675054	3,17
208284_x_at	GGT1	2678	NM_0134	3,14
235773_at	ZIK1	284307	AW02929	3,14
37028_at	PPP1R15A	23645	U83981	3,09
239359_at	MARCH-X	441061	AA383208	3,09
206343_s_at	NRG1	3084	NM_0139	3,08
242070_at	LOC72848	728485	AI014470	3,08
32699_s_at	PVR	5817	X64116	3,05
225354_s_at	SH3BGRL	83699	AL035700	3,03
209921_at	SLC7A11	23657	AB040875	3,02
221667_s_at	HSPB8	26353	AF133207	3,02
242450_at	RGMB	285704	AW00471	3,01
232099_at	PCDHB16	57717	AB04684	2,99
205659_at	HDAC9	9734	NM_0147	2,98
215071_s_at	HIST1H2A	8334	AL353759	2,98
205608_s_at	ANGPT1	284	U83508	2,97
242429_at	ZNF567	163081	BF435183	2,94
205210_at	TGFBRAP1	9392	NM_0042	2,93
226118_at	CENPO	79172	BE326728	2,92
210793_s_at	NUP98	4928	U41815	2,92
232315_at	LOC40071	400713	AU14971	2,92
222686_s_at	FLJ11151	55313	AL572407	2,89
235287_at	CDK6	1021	AW19270	2,85
218900_at	CNNM4	26504	NM_0201	2,84
234300_s_at	ZFP28	140612	AF226995	2,83
201107_s_at	THBS1	7057	AI812030	2,82
227196_at	RHPN2	85415	BG05498	2,80
1558815_at	SORBS2	8470	BC035329	2,80
1552680_a_at	CASC5	57082	NM_0203	2,79
223800_s_at	LIMS3	96626	AF288404	2,78
228104_at	PLXNA4A	57671	AL117427	2,76
1569190_at	SCLT1	132320	BC014677	2,76
243529_at	MARS2	92935	BE542381	2,73
226702_at	LOC12960	129607	AI742057	2,72
202083_s_at	SEC14L1	6397	AI017770	2,71
1554757_a_at	INPP5A	3632	AF273055	2,70
238686_at	FBXO3	26273	AA130258	2,70
235121_at	ZNF542	147947	BE966943	2,69
235818_at	VSTM1	284415	AI498747	2,69
212420_at	ELF1	1997	AL559590	2,68
242761_s_at	ZNF420	147923	AI061288	2,68
214600_at	TEAD1	7003	AW77193	2,65
1554396_at	UEVLD	55293	BC011011	2,65
1554433_a_at	ZNF146	7705	BC005154	2,65
229744_at	SSFA2	6744	AL556611	2,63
206084_at	PTPRR	5801	NM_0028	2,63
208017_s_at	MCF2	4168	NM_0053	2,62
222227_at	FLJ20840	n/a	AK000847	2,62
202082_s_at	SEC14L1	6397	AV748469	2,62
1555355_a_at	ETS1	2113	BC017314	2,61
202014_at	PPP1R15A	23645	NM_0143	2,61
203288_at	KIAA0355	9710	NM_0146	2,61
217678_at	SLC7A11	23657	AA488687	2,60
207131_x_at	GGT1	2678	NM_0134	2,60
201721_s_at	LAPTM5	7805	NM_0067	2,59
220030_at	STYK1	55359	NM_0184	2,59

227611_at	TARSL2	123283	AA442856	2,58
235648_at	ZNF567	163081	AA742659	2,58
243000_at	CDK6	1021	AW19476	2,58
223710_at	CCL26	10344	AF096296	2,58
237061_at	ZNF347	84671	AI810186	2,57
1555007_s_at	WDR66	144406	BC03623:	2,57
234799_at	ADARB1	104	AK02722	2,56
219848_s_at	ZNF432	9668	NM_0146	2,56
228825_at	LTB4DH	22949	BE566894	2,55
202669_s_at	EFNB2	1948	U16797	2,54
1553962_s_at	RHOB	388	BI668074	2,54
218849_s_at	PPP1R13L	10848	NM_0066	2,53
236620_at	RIF1	55183	AU15084:	2,53
58916_at	KCTD14	65987	AI672101	2,52
211417_x_at	GGT1	2678	L20493	2,52
202668_at	EFNB2	1948	BF00167(	2,51
1555793_a_at	ZNF545	284406	AL834267	2,50
242139_s_at	n/a	n/a	AA635618	2,50
239787_at	KCTD4	386618	N52767	2,50
237290_at	n/a	n/a	AW13887	2,50
232080_at	HECW2	57520	AL390186	2,50
202124_s_at	TRAK2	66008	AV70525:	2,49
230788_at	GCNT2	2651	BF059748	2,47
207090_x_at	ZFP30	22835	NM_0148	2,44
213765_at	MFAP5	8076	AW66589	2,44
227920_at	KIAA1553	57673	AV700885	2,44
207198_s_at	LIMS1	3987	NM_0049	2,44
228450_at	PLEKHA7	144100	AA75886:	2,43
238436_s_at	ZNF805	390980	AV726376	2,43
239237_at	n/a	n/a	AI798822	2,43
239835_at	KBTBD8	84541	AA669114	2,43
225614_at	SAAL1	113174	AI815207	2,42
219495_s_at	ZNF180	7733	NM_0132	2,41
228920_at	ZNF260	339324	BE328273	2,40
211975_at	ZNF289	84364	BE299671	2,40
206487_at	UNC84A	23353	NM_0251	2,40
219014_at	PLAC8	51316	NM_0166	2,37
214963_at	NUP160	23279	AK026236	2,36
1554400_at	TCTE3	6991	AF519569	2,35
220386_s_at	EML4	27436	NM_0190	2,35
243188_at	ZNF283	284349	AI753038	2,34
221696_s_at	STYK1	55359	AF251059	2,34
204180_s_at	ZBTB43	23099	AI745225	2,34
225863_s_at	C19orf12	83636	AL568826	2,33
206371_at	FOLR3	2352	NM_0008	2,33
228229_at	ZNF526	116115	BF512165	2,33
207740_s_at	NUP62	23636	NM_0123	2,33
244462_at	ZNF224	7767	AA81198:	2,32
212336_at	EPB41L1	2036	AA91271:	2,31
232228_at	ZNF530	348327	AA73768:	2,31
203833_s_at	TGOLN2	10618	BF061845	2,30
209305_s_at	GADD45B	4616	AF078077	2,30
202672_s_at	ATF3	467	NM_0016	2,30
242705_x_at	n/a	n/a	AI188104	2,29
217223_s_at	BCR	613	U07000	2,28
1558700_s_at	ZNF260	339324	BC042676	2,28

241418_at	LOC34488	344887	AI819386	2,28
223765_s_at	KBTBD4	55709	AF151086	2,28
239233_at	CCDC88A	55704	AA74461	2,28
207666_x_at	SSX3	10214	NM_0210	2,27
215509_s_at	BUB1	699	AL137654	2,27
216689_x_at	ARHGAP1	392	U62794	2,26
238716_at	n/a	n/a	AL527331	2,26
205658_s_at	SNAPC4	6621	NM_0030	2,24
1553718_at	ZNF548	147694	NM_1529	2,23
1555058_a_at	LPGAT1	9926	BC03462	2,23
236429_at	n/a	n/a	AI831874	2,23
206706_at	NTF3	4908	NM_0025	2,22
211136_s_at	CLPTM1	1209	BC004865	2,22
222599_s_at	NAV2	89797	AU13760	2,22
205832_at	CPA4	51200	NM_0163	2,22
236946_at	n/a	n/a	AI220134	2,21
220921_at	SPANXB1	64694 ///	NM_0134	2,21
215603_x_at	GGT1 ///	2678 ///	AI344075	2,20
228086_at	STK33	65975	AI703417	2,20
228882_at	TUB	7275	AL042088	2,20
214443_at	PVR	5817	NM_0065	2,20
1555733_s_at	AP1S3	130340	AF393369	2,19
1554018_at	GPNMB	10457	BC011595	2,19
242787_at	n/a	n/a	AI924134	2,19
212133_at	NIPA2	81614	AI681536	2,18
214975_s_at	MTMR1	8776	AK001816	2,17
203895_at	PLCB4	5332	AL535113	2,17
221014_s_at	RAB33B	83452	NM_0312	2,16
213447_at	IPW	3653	AI672541	2,16
201615_x_at	CALD1	800	AI685060	2,16
215856_at	SIGLEC15	284266	AK02583	2,16
228713_s_at	HSD17B1	51171	AI742586	2,15
204433_s_at	SPATA2	9825	U28164	2,15
205425_at	HIP1	3092	NM_0053	2,15
229845_at	MAPKAP1	79109	BF939919	2,15
243194_at	ZNF551	90233	BF438407	2,15
204857_at	MAD1L1	8379	NM_0035	2,15
206155_at	ABCC2	1244	NM_0003	2,14
1564378_a_at	n/a	n/a	AK02510	2,14
227045_at	ZNF614	80110	AI087872	2,13
202570_s_at	DLGAP4	22839	BF346592	2,13
215881_x_at	LOC6521	10214 ///	BF18427	2,13
213124_at	ZNF473	25888	BG53880	2,12
222028_at	ZNF45	7596	AI967981	2,12
201411_s_at	PLEKHB2	55041	NM_0179	2,12
232961_at	LOC26010	26010	AU14816	2,12
215739_s_at	TUBGCP3	10426	AJ003062	2,11
206569_at	IL24	11009	NM_0068	2,10
229412_at	TAF8	129685	AA64125	2,10
207034_s_at	GLI2	2736	NM_0303	2,10
205020_s_at	ARL4A	10124	NM_0057	2,10
201720_s_at	LAPTM5	7805	AI589086	2,09
217627_at	ZNF573	126231	BE515346	2,09
1554378_a_at	PDE1C	5137	BC022479	2,09
1555154_a_at	QKI	9444	AF142421	2,08
203123_s_at	SLC11A2	4891	AU154469	2,08

209287_s_at	CDC42EP	10602	AF10485	2,08
201537_s_at	DUSP3	1845	BC00268	2,07
234001_s_at	ARFGAP1	55738	AL13774	2,07
221974_at	IPW	3653	AW77074	2,07
1557128_at	FAM111B	374393	AA96084	2,06
219474_at	C3orf52	79669	NM_0246	2,06
210675_s_at	PTPRR	5801	U77917	2,06
216283_s_at	PVR	5817	X64116	2,05
224848_at	CDK6	1021	AA92206	2,05
208579_x_at	H2BFS	54145	NM_0174	2,05
218972_at	TTC17	55761	NM_0182	2,04
224913_s_at	TIMM50	92609	AA87782	2,04
206182_at	ZNF134	7693	NM_0034	2,04
1557267_s_at	LOC2849	284952	BF51323	2,04
229221_at	CD44	960	BE46702	2,03
214723_x_at	KIAA1641	57730	AB04686	2,03
213059_at	CREB3L1	90993	AF05500	2,03
211681_s_at	PDLIM5	10611	AF11670	2,03
201538_s_at	DUSP3	1845	NM_0040	2,02
238944_at	n/a	n/a	AI393706	2,01
230123_at	NECAP2	55707	AI608836	2,01
204544_at	HPS5	11234	NM_0072	2,01
209035_at	MDK	4192	M69148	2,00
205082_s_at	AOX1	316	AB04669	2,00
204642_at	EDG1	1901	NM_0014	2,00
204614_at	SERPINB	5055	NM_0025	1,99
202453_s_at	GTF2H1	2965	NM_0053	1,99
1553993_s_at	MED25	81857	BC02431	1,99
1561853_a_at	IL23R	149233	BC01682	1,98
212238_at	ASXL1	171023	AL11751	1,98
230560_at	STXBP6	29091	N21096	1,97
229274_at	GNAS	2778	AI693143	1,97
225296_at	ZNF317	57693	AB04680	1,97
209238_at	STX3	6809	BE96692	1,97
1556911_at	n/a	n/a	BC04148	1,96
209462_at	APLP1	333	U48437	1,96
202912_at	ADM	133	NM_0011	1,95
209655_s_at	TMEM47	83604	AI803181	1,94
215195_at	PRKCA	5578	AF03559	1,93
209380_s_at	ABCC5	10057	AF14607	1,93
228652_at	ZNF776	284309	AI279532	1,93
211981_at	COL4A1	1282	NM_0018	1,93
202368_s_at	TRAM2	9697	AI986461	1,93
1562240_at	PLXNA4A	57671	AB04677	1,93
235476_at	TRIM59	286827	AW18245	1,92
208879_x_at	PRPF6	24148	BG46903	1,92
225590_at	SH3RF1	57630	AI686957	1,92
206170_at	ADRB2	154	NM_0000	1,92
230112_at	MARCH-4	57574	AB03782	1,92
233445_at	n/a	n/a	AK02204	1,92
1561631_at	n/a	n/a	AF14741	1,91
227510_x_at	PRO1073	29005	AL03791	1,91
210299_s_at	FHL1	2273	AF06300	1,91
204236_at	FLI1	2313	NM_0020	1,91
219387_at	CCDC88A	55704	NM_0175	1,91
235484_at	PTAR1	375743	BE89288	1,91

223707_at	MGC1085	84736	BC00428	1,91
200787_s_at	PEA15	8682	BC00242	1,90
222455_s_at	PARVA	55742	AF237771	1,90
213030_s_at	PLXNA2	5362	AI688418	1,90
1569366_a_at	ZNF569	148266	BC03873	1,90
235882_at	n/a	n/a	BF115777	1,89
241820_at	RIF1	55183	BF666241	1,89
213909_at	LRRC15	131578	AU14779	1,89
209021_x_at	KIAA0652	9776	BC00133	1,89
202436_s_at	CYP1B1	1545	AU14485	1,89
227827_at	n/a	n/a	AW13814	1,88
202149_at	NEDD9	4739	AL136139	1,88
1558747_at	SMCHD1	23347	AA33650	1,88
235548_at	APCDD1L	164284	BG32659	1,88
204875_s_at	GMDS	2762	NM_0015	1,88
205264_at	CD3EAP	10849	NM_0120	1,87
211423_s_at	SC5DL	6309	D85181	1,87
209516_at	SMYD5	10322	U50383	1,87
203702_s_at	TTLL4	9654	AL043927	1,86
204696_s_at	CDC25A	993	NM_0017	1,86
209919_x_at	GGT1	2678	L20490	1,86
231182_at	WIPF1	7456	BF446719	1,86
213248_at	LOC73010	730101	AL57702	1,86
221563_at	DUSP10	11221	N36770	1,86
1554095_at	RBM33	155435	BC01192	1,86
223117_s_at	USP47	55031	AW02509	1,85
217981_s_at	FXC1	26515	NM_0121	1,85
1553719_s_at	ZNF548	147694	NM_1529	1,85
227718_at	PURB	5814	BF33779	1,84
204233_s_at	CHKA	1119	AI991328	1,84
200769_s_at	MAT2A	4144	NM_0059	1,84
205670_at	GAL3ST1	9514	NM_0048	1,84
202726_at	LIG1	3978	NM_0002	1,83
207992_s_at	AMPD3	272	NM_0004	1,83
1554479_a_at	CARD8	22900	AF51165	1,83
209015_s_at	DNAJB6	10049	BC00244	1,83
218939_at	LETM1	3954	NM_0123	1,83
215617_at	n/a	n/a	AU14571	1,83
206950_at	SCN9A	6335	NM_0029	1,83
232198_at	n/a	n/a	BF509125	1,83
205862_at	GREB1	9687	NM_0146	1,83
1558836_at	n/a	n/a	BQ02449	1,82
1555772_a_at	CDC25A	993	AY13758	1,82
228370_at	SNRPN	6638	BF11487	1,82
1553221_at	ZNF583	147949	NM_1524	1,81
232184_at	ALS2	57679	AK02302	1,81
221676_s_at	CORO1C	23603	BC00234	1,81
203642_s_at	COBLL1	22837	NM_0149	1,81
202084_s_at	SEC14L1	6397	NM_0030	1,80
208490_x_at	HIST1H2B	8343	NM_0035	1,80
201101_s_at	BCLAF1	9774	BE96337	1,80
204589_at	NUAK1	9891	NM_0148	1,79
237554_at	n/a	n/a	BE34830	1,79
215151_at	DOCK10	55619	AB01459	1,79
201917_s_at	SLC25A36	55186	AI694452	1,78
208016_s_at	AGTR1	185	NM_0048	1,78

217889_s_at	CYBRD1	79901	NM_0248	1,78
209535_s_at	n/a	n/a	AF127481	1,78
209314_s_at	HBS1L	10767	AK02425[illegible]	1,78
201616_s_at	CALD1	800	AL577531	1,78
205401_at	AGPS	8540	NM_0036	1,78
221765_at	UGCG	7357	AI378044	1,77
1554661_s_at	C1orf71	163882	BC03620[illegible]	1,77
221734_at	PRRC1	133619	BE32831[illegible]	1,77
235399_at	n/a	n/a	AA68249[illegible]	1,76
205095_s_at	ATP6V0A[illegible]	535	NM_0051	1,76
201540_at	FHL1	2273	NM_0014	1,76
231879_at	COL12A1	1303	AL096771	1,76
205357_s_at	AGTR1	185	NM_0006	1,76
219228_at	ZNF331	55422	NM_0185	1,76
215821_x_at	PSG3	5671	R32065	1,76
240239_at	ZNF566	84924	N63953	1,75
221903_s_at	CYLD	1540	BE04644[illegible]	1,75
223506_at	ZC3H8	84524	AF334161	1,75
226437_at	YIF1B	90522	AI079540	1,75
64488_at	n/a	n/a	AW00309	1,75
228797_at	n/a	n/a	AI140917	1,74
216869_at	PDE1C	5137	U40372	1,74
1555450_a_at	NARG1L	79612	BC03231[illegible]	1,74
219711_at	ZNF586	54807	NM_0176	1,74
225928_at	VTI1B	10490	AI984620	1,74
238831_at	n/a	n/a	BF11467[illegible]	1,74
217884_at	NAT10	55226	NM_0246	1,74
232982_at	AP1GBP1	11276	AF09092[illegible]	1,73
227223_at	LOC6431[illegible]	643167 /[illegible]	BE46617[illegible]	1,73
205387_s_at	CGB /// C	1082 /// [illegible]	NM_0007	1,73
1552946_at	ZNF114	163071	NM_1536	1,73
228641_at	CARD8	22900	BF33838[illegible]	1,73
226602_s_at	BCR /// F	400892 /[illegible]	T30183	1,73

	Down-regulated genes in T vs P			
Probe set	Gene	Gene ID	Public ID	Fold change (log2 ratio)
206336_at	CXCL6	6372	NM_0029	-8,97
210982_s_at	HLA-DRA	3122	M60333	-8,70
211990_at	HLA-DPA1	3113	M27487	-8,02
208894_at	HLA-DRA	3122	M60334	-7,61
219049_at	ChGn	55790	NM_0183	-6,50
223551_at	PKIB	5570	AF225513	-6,40
204439_at	IFI44L	10964	NM_0068	-6,15
202202_s_at	LAMA4	3910	NM_0022	-6,15
234973_at	SLC38A5	92745	BG32563(	-5,96
211991_s_at	HLA-DPA1	3113	M27487	-5,88
204670_x_at	HLA-DRB1	3123; 73(	NM_0021	-5,59
203029_s_at	PTPRN2	5799	NM_0028	-5,45
209312_x_at	HLA-DRB1	3123; 73(	U65585	-5,44
227058_at	C13orf33	84935	AW08473	-5,39
229802_at	n/a	n/a	AA147884	-5,35
39402_at	IL1B	3553	M15330	-5,25
213506_at	F2RL1	2150	BE965369	-5,23
209619_at	CD74	972	K01144	-5,18
212657_s_at	IL1RN	3557	U65590	-5,15
205919_at	HBE1	3046	NM_0053	-5,08
205067_at	IL1B	3553	NM_0005	-5,04
210367_s_at	PTGES	9536	AF010316	-5,01
212951_at	GPR116	221395	N95226	-5,00
221901_at	KIAA1644	85352	BF516072	-4,97
213537_at	HLA-DPA1	3113	AI128225	-4,96
238332_at	ANKRD29	147463	AI307802	-4,93
211506_s_at	IL8	3576	AF043337	-4,84
224989_at	n/a	n/a	AI824013	-4,81
205576_at	SERPIND1	3053	NM_0001	-4,76
52837_at	KIAA1644	85352	AL047020	-4,63
215193_x_at	HLA-DRB1	3123; 31	AJ297586	-4,54
204463_s_at	EDNRA	1909	AU11888	-4,49
208306_x_at	HLA-DRB1	3123	NM_0219	-4,43
202859_x_at	IL8	3576	NM_0005	-4,40
207173_x_at	CDH11	1009	D21254	-4,31
226722_at	FAM20C	56975	BE874872	-4,27
219908_at	DKK2	27123	NM_0144	-4,08
223093_at	ANKH	56172	T99215	-4,07
231120_x_at	PKIB	5570	AL569326	-4,06
212912_at	RPS6KA2	6196	AI992251	-4,03
201417_at	SOX4	6659	AL136179	-4,03
230383_x_at	n/a	n/a	AA133285	-4,00
212148_at	PBX1	5087	AL049381	-3,98
208650_s_at	CD24	934	BG32786	-3,91
212671_s_at	HLA-DQA	3117; 31	BG397856	-3,90
226777_at	ADAM12	8038	AA14793	-3,89
1568983_a_at	n/a	n/a	BI547087	-3,88
230109_at	PDE7B	27115	AI638433	-3,88
227052_at	n/a	n/a	AI810669	-3,87
224463_s_at	C11orf70	85016	BC006128	-3,83
217478_s_at	HLA-DMA	3108	X76775	-3,81
213338_at	TMEM158	25907	BF062629	-3,79
206513_at	AIM2	9447	NM_0048	-3,77
202016_at	MEST	4232	NM_0024	-3,76

210942_s_at	ST3GAL6	10402	AB02291	-3,71
223744_s_at	SIAE	54414	AF30079	-3,71
226301_at	C6orf192	116843	AV72907	-3,68
243582_at	SH3RF2	153769	AW08263	-3,64
209771_x_at	CD24	934	AA76118	-3,64
214078_at	n/a	n/a	AF070581	-3,63
203083_at	THBS2	7058	NM_0032	-3,63
201348_at	GPX3	2878	NM_0020	-3,60
203504_s_at	ABCA1	19	NM_0055	-3,55
229414_at	PITPNC1	26207	AI676095	-3,54
228501_at	GALNTL2	117248	BF05534	-3,52
209351_at	KRT14	3861	BC00269	-3,50
205856_at	SLC14A1	6563	NM_0158	-3,46
236277_at	n/a	n/a	H23551	-3,45
226258_at	AMN1	196394	BG03189	-3,44
217562_at	FAM5C	339479	BF58952	-3,43
220330_s_at	SAMSN1	64092	NM_0221	-3,42
238478_at	BNC2	54796	H97386	-3,41
266_s_at	CD24	934	L33930	-3,41
236361_at	GALNTL2	117248	BF43237	-3,40
221797_at	LOC3392	339229	AY00712	-3,39
229308_at	n/a	n/a	AW27379	-3,36
224990_at	C4orf34	201895	BE97272	-3,35
212950_at	GPR116	221395	BF94149	-3,35
203180_at	ALDH1A3	220	NM_0006	-3,33
218807_at	VAV3	10451	NM_0061	-3,32
1554067_at	FLJ32549	144577	BC03624	-3,31
213716_s_at	SECTM1	6398	BF93967	-3,25
204464_s_at	EDNRA	1909	NM_0019	-3,24
228661_s_at	n/a	n/a	AI768374	-3,22
227059_at	GPC6	10082	AI651255	-3,20
218983_at	C1RL	51279	NM_0165	-3,19
213488_at	SNED1	25992	N73970	-3,17
1556935_at	n/a	n/a	AF08583	-3,13
221024_s_at	SLC2A10	81031	NM_0307	-3,13
219799_s_at	DHRS9	10170	NM_0057	-3,13
228708_at	RAB27B	5874	BF43838	-3,13
219182_at	FLJ22167	79583	NM_0245	-3,10
204417_at	GALC	2581	NM_0001	-3,10
208747_s_at	C1S	716	M18767	-3,10
238504_at	C6orf57	135154	AA52102	-3,09
212998_x_at	HLA-DQB	3119	AI583173	-3,09
207761_s_at	METTL7A	25840	NM_0140	-3,09
218162_at	OLFML3	56944	NM_0201	-3,08
203903_s_at	HEPH	9843	NM_0147	-3,07
203126_at	IMPA2	3613	NM_0142	-3,07
209894_at	LEPR	3953	U50748	-3,07
209763_at	CHRDL1	91851	AL04917	-3,06
206481_s_at	LDB2	9079	NM_0012	-3,06
204491_at	PDE4D	5144	R40917	-3,03
219771_at	TBC1D8B	54885	NM_0177	-3,03
202037_s_at	SFRP1	6422	NM_0030	-3,02
229544_at	n/a	n/a	AI690169	-3,01
229872_s_at	LOC6424	642441;	AA53265	-3,00
235274_at	n/a	n/a	AA74063	-2,96
228155_at	C10orf58	84293	BF51238	-2,95

202036_s_at	SFRP1	6422	AF017987	-2,95
207713_s_at	RBCK1	10616	NM_0064	-2,94
203543_s_at	KLF9	687	NM_0012	-2,93
205122_at	TMEFF1	8577	BF439316	-2,91
204273_at	EDNRB	1910	NM_0001	-2,91
228176_at	EDG3	1903	AA534817	-2,89
205302_at	IGFBP1	3484	NM_0005	-2,88
235428_at	n/a	n/a	H78106	-2,87
222754_at	TRNT1	51095	BE552215	-2,87
201137_s_at	HLA-DPB1	3115	NM_0021	-2,87
204470_at	CXCL1	2919	NM_0015	-2,87
230722_at	BNC2	54796	AI377043	-2,87
213524_s_at	G0S2	50486	NM_0157	-2,85
211161_s_at	COL3A1	1281	AF130082	-2,84
226800_at	KIAA1799	84455	AL109925	-2,83
213032_at	NFIB	4781	AI186739	-2,82
201150_s_at	TIMP3	7078	NM_0003	-2,82
228606_at	MGC3321	255758	AW01671	-2,81
238649_at	PITPNC1	26207	AA815089	-2,81
1556329_a_at	n/a	n/a	BC042378	-2,80
1558801_at	n/a	n/a	AK055769	-2,80
227238_at	MUC15	143662	W93847	-2,80
222614_at	RWDD2B	10069	AF212232	-2,79
224516_s_at	CXXC5	51523	BC006428	-2,78
211896_s_at	DCN	1634	AF138302	-2,76
203505_at	ABCA1	19	AF285167	-2,75
216379_x_at	CD24	934	AK000168	-2,75
204457_s_at	GAS1	2619	NM_0020	-2,74
231979_at	n/a	n/a	AU155091	-2,73
220076_at	ANKH	56172	NM_0198	-2,71
221787_at	C6orf120	387263	BF431618	-2,70
218820_at	C14orf132	56967	NM_0202	-2,69
207017_at	RAB27B	5874	NM_0041	-2,67
205234_at	SLC16A4	9122	NM_0046	-2,67
209335_at	DCN	1634	AI281593	-2,67
228909_at	C21orf86	257103	AW13155	-2,65
236088_at	NTNG1	22854	AV723308	-2,65
219911_s_at	SLCO4A1	28231	NM_0163	-2,64
239370_at	n/a	n/a	AW08198	-2,64
207069_s_at	SMAD6	4091	NM_0055	-2,64
202957_at	HCLS1	3059	NM_0053	-2,64
222996_s_at	CXXC5	51523	BC002490	-2,64
215076_s_at	COL3A1	1281	AU144167	-2,63
203030_s_at	PTPRN2	5799	AF007555	-2,63
1553778_at	WBSCR27	155368	NM_1525	-2,61
228185_at	ZNF25	219749	N32599	-2,60
209480_at	HLA-DQB	3119	M16276	-2,60
218773_s_at	MSRB2	22921	NM_0122	-2,58
213587_s_at	ATP6V0E2	155066	AI884867	-2,58
212423_at	C10orf56	219654	AK024784	-2,58
222288_at	n/a	n/a	AI004009	-2,57
210534_s_at	EPPB9	27077	BC002944	-2,57
218980_at	FHOD3	80206	NM_0251	-2,57
221864_at	ORAI3	93129	AW51746	-2,56
203932_at	HLA-DMB	3109	NM_0021	-2,56
242794_at	MAML3	55534	AI569476	-2,56

209469_at	GPM6A	2823	BF939489	-2,56
213355_at	ST3GAL6	10402	AI989567	-2,55
214920_at	THSD7A	221981	R33964	-2,55
203017_s_at	SSX2IP	117178	R52678	-2,55
1556555_at	n/a	n/a	AI473891	-2,55
212444_at	n/a	n/a	AA156240	-2,54
202273_at	PDGFRB	5159	NM_0026	-2,54
221031_s_at	APOLD1	81575	NM_0308	-2,54
223952_x_at	DHRS9	10170	AF240698	-2,54
227074_at	n/a	n/a	AA524669	-2,53
206785_s_at	KLRC1; K	3821; 382	NM_0022	-2,53
212419_at	C10orf56	219654	AA131324	-2,51
206026_s_at	TNFAIP6	7130	NM_0071	-2,51
205640_at	ALDH3B1	221	NM_0006	-2,51
202990_at	PYGL	5836	NM_0028	-2,50
230054_at	PRRT1	80863	AW13449	-2,49
223796_at	CNTNAP3	389734;	AF333769	-2,49
229152_at	C4orf7	260436	AI718421	-2,49
207388_s_at	PTGES	9536	NM_0048	-2,49
212913_at	C6orf26;	401251;	BE674960	-2,49
211813_x_at	DCN	1634	AF138303	-2,48
48825_at	ING4	51147	AA887083	-2,48
206766_at	ITGA10	8515	AF112345	-2,48
242358_at	n/a	n/a	AW02465	-2,48
226725_at	SLFN5	162394	AI435399	-2,47
221019_s_at	COLEC12	81035	NM_0307	-2,46
225275_at	EDIL3	10085	AA053711	-2,46
209016_s_at	KRT7	3855	BC002700	-2,45
213831_at	HLA-DQA	3117	X00452	-2,45
213392_at	IQCK	124152	AW07022	-2,44
213493_at	SNED1	25992	BF509657	-2,42
223478_at	TIMM8B	26521	AF165967	-2,42
225842_at	PHLDA1	22822	AK026181	-2,42
223204_at	C4orf18	51313	AF260333	-2,42
1558692_at	C1orf85	112770	AW09018	-2,42
223843_at	SCARA3	51435	AB007830	-2,41
1566342_at	n/a	n/a	R34841	-2,41
212070_at	GPR56	9289	AL554008	-2,40
201655_s_at	HSPG2	3339	M85289	-2,38
226134_s_at	n/a	n/a	AI978754	-2,38
219147_s_at	C9orf95	54981	NM_0178	-2,38
200832_s_at	SCD	6319	AB032261	-2,38
209277_at	TFPI2	7980	AL574096	-2,38
213698_at	ZMYM6	9204	AI805560	-2,38
211760_s_at	VAMP4	8674	BC005974	-2,38
235521_at	HOXA3	3200	AW13798	-2,37
1556033_at	n/a	n/a	BQ187042	-2,36
229011_at	n/a	n/a	AA150501	-2,35
213621_s_at	GUK1	2987	AW18289	-2,35
201893_x_at	DCN	1634	AF138300	-2,35
212268_at	SERPINB1	1992	NM_0306	-2,34
203763_at	DYNC2LI1	51626	NM_0160	-2,34
64900_at	FLJ22167	79583	AA401703	-2,34
229070_at	C6orf105	84830	AA470369	-2,34
201842_s_at	EFEMP1	2202	AI826799	-2,34
206027_at	S100A3	6274	NM_0029	-2,33

221491_x_at	n/a	3123; 31[illegible]	AA80705[illegible]	-2,33
202218_s_at	FADS2	9415	NM_0042	-2,33
205266_at	LIF	3976	NM_0023	-2,33
242005_at	n/a	n/a	BE87742[illegible]	-2,32
238178_at	n/a	n/a	BF11026[illegible]	-2,32
221911_at	ETV1	2115	BE88159[illegible]	-2,32
212192_at	KCTD12	115207	AI718937	-2,32
235889_at	n/a	n/a	AI825987	-2,31
225240_s_at	MSI2	124540	BE22002[illegible]	-2,31
225534_at	C8orf40	114926	AV71134[illegible]	-2,31
202411_at	IFI27	3429	NM_0055	-2,31
214764_at	RRP15	51018	AW02916	-2,30
227094_at	DHTKD1	55526	AI934407	-2,28
223094_s_at	ANKH	56172	AF27475[illegible]	-2,28
220387_s_at	HHLA3	11147	NM_0070	-2,27
201852_x_at	COL3A1	1281	AI813758	-2,27
223179_at	YPEL3	83719	BC00500[illegible]	-2,27
230866_at	CYSLTR1	10800	BE54954[illegible]	-2,26
237292_at	n/a	n/a	BE66970[illegible]	-2,26
218953_s_at	PCYOX1L	78991	NM_0240	-2,25
214657_s_at	TncRNA	283131	AU13497[illegible]	-2,25
204035_at	SCG2	7857	NM_0034	-2,24
226847_at	FST	10468	BF43817[illegible]	-2,24
244881_at	LMLN	89782	AA62905[illegible]	-2,23
243824_at	n/a	n/a	AA52108[illegible]	-2,23
233329_s_at	KRCC1	51315	AK02598[illegible]	-2,23
224443_at	C1orf97	84791	BC00599[illegible]	-2,22
205682_x_at	APOM	55937	NM_0191	-2,22
216235_s_at	EDNRA	1909	S81545	-2,20
221606_s_at	NSBP1	79366	BC00534[illegible]	-2,20
204821_at	BTN3A3	10384	NM_0069	-2,20
232504_at	miR-146a	406938	AL389942	-2,20
201843_s_at	EFEMP1	2202	NM_0041	-2,19
214151_s_at	CCPG1	9236	AU14424[illegible]	-2,18
223041_at	CD99L2	83692	AL13658[illegible]	-2,18
203542_s_at	KLF9	687	AI690205	-2,17
228737_at	TOX2	84969	AA21190[illegible]	-2,17
206924_at	IL11	3589	NM_0006	-2,16
202250_s_at	WDR42A	50717	NM_0157	-2,16
211654_x_at	HLA-DQB	3119; 65[illegible]	M17565	-2,15
214954_at	SUSD5	26032	BF977837	-2,15
208906_at	BSCL2; H	221092; [illegible]	BC00491[illegible]	-2,14
1568745_at	hCG_165[illegible]	646268	BC02959[illegible]	-2,14
1563246_at	n/a	n/a	BC03819[illegible]	-2,14
203789_s_at	SEMA3C	10512	NM_0063	-2,14
205104_at	SNPH	9751	NM_0147	-2,12
218175_at	CCDC92	80212	NM_0251	-2,12
205110_s_at	FGF13	2258	NM_0041	-2,11
228316_at	FLJ31438	130162	AA90547[illegible]	-2,11
217763_s_at	RAB31	11031	NM_0068	-2,11
228700_at	n/a	n/a	AA06360[illegible]	-2,11
226849_at	DENND1A	57706	AB04682[illegible]	-2,10
218204_s_at	FYCO1	79443	NM_0245	-2,10
235956_at	KIAA1377	57562	AI797063	-2,10
211742_s_at	EVI2B	2124	BC00592[illegible]	-2,09
202035_s_at	SFRP1	6422	AI332407	-2,09

1553118_at	THEM4	117145	NM_0530	-2,08
225237_s_at	MSI2	124540	BF435123	-2,08
203882_at	ISGF3G	10379	NM_0060	-2,07
242239_at	n/a	n/a	AW97088	-2,07
205992_s_at	IL15	3600	NM_0005	-2,07
211799_x_at	HLA-C	3107	U62824	-2,07
203143_s_at	KIAA0040	9674	T79953	-2,07
202321_at	GGPS1	9453	AW29950	-2,07
209278_s_at	TFPI2	7980	L27624	-2,06
224763_at	RPL37	6167	BF724210	-2,06
213222_at	PLCB1	23236	AL049593	-2,06
222784_at	SMOC1	64093	AJ249900	-2,06
226682_at	LOC28366	283666	AW00618	-2,06
201708_s_at	NIPSNAP1	8508	AW08337	-2,05
234929_s_at	SPATA7	55812	AF144488	-2,05
220272_at	BNC2	54796	NM_0176	-2,05
238063_at	TMEM154	201799	AA806283	-2,05
1562013_a_at	n/a	n/a	AK074453	-2,05
204480_s_at	C9orf16	79095	NM_0241	-2,04
203414_at	MMD	23531	NM_0123	-2,04
232138_at	MBNL2	10150	AW27691	-2,04
242273_at	n/a	n/a	AA747287	-2,04
1555888_at	UBR5	51366	AK095151	-2,04
204719_at	ABCA8	10351	NM_0071	-2,04
207850_at	CXCL3	2921	NM_0020	-2,03
227688_at	LRCH2	57631	AK022128	-2,03
231001_at	FIBIN	387758	AI755024	-2,03
209531_at	GSTZ1	2954	BC001453	-2,03
244015_at	n/a	n/a	AA704163	-2,03
202820_at	AHR	196	NM_0016	-2,03
1557813_at	n/a	n/a	BF724621	-2,02
237563_s_at	LOC44073	440731	AI286239	-2,02
213496_at	LPPR4	9890	AW59256	-2,02
222772_at	MYEF2	50804	BG179854	-2,02
238919_at	n/a	n/a	R49295	-2,02
211911_x_at	HLA-B	3106	L07950	-2,01
229145_at	C10orf104	119504	AA541762	-2,00
243438_at	PDE7B	27115	BE968570	-2,00
226237_at	n/a	n/a	AL359062	-2,00
50374_at	LOC33922	339229	AA150503	-2,00
221892_at	H6PD	9563	AK024548	-1,99
235704_at	DAZAP2	9802	AI307251	-1,99
232382_s_at	PCMTD1	115294	BE150929	-1,99
232726_at	n/a	n/a	AK024956	-1,99
229465_s_at	n/a	n/a	BF433071	-1,99
238133_at	n/a	n/a	AW05159	-1,98
219100_at	OBFC1	79991	NM_0249	-1,98
210115_at	RPL39L	116832	L05096	-1,98
226736_at	CHURC1	91612	BE568660	-1,97
202472_at	MPI	4351	NM_0024	-1,97
226779_at	n/a	n/a	BF432857	-1,97
225525_at	CTA-221G	85379	AB051458	-1,97
226040_at	n/a	n/a	BE856302	-1,96
236453_at	LOC44144	441440	AW24315	-1,96
235613_at	n/a	n/a	BF476152	-1,96
224435_at	C10orf58	84293	BC005871	-1,96

205280_at	GLRB	2743	NM_0008	-1,95
203919_at	TCEA2	6919	NM_0031	-1,95
1555852_at	n/a	n/a	AI375915	-1,95
206833_s_at	ACYP2	98	NM_0011	-1,95
229201_at	n/a	n/a	AW04465	-1,93
226762_at	PURB	5814	AV70909	-1,93
212182_at	NUDT4; N	11163; 4	AB00795	-1,93
218436_at	SIL1	64374	NM_0224	-1,93
226361_at	TMEM42	131616	BF05609	-1,93
238067_at	TBC1D8B	54885	AW17243	-1,92
238050_at	n/a	n/a	R94785	-1,91
214022_s_at	IFITM1	8519	AA74910	-1,90
218976_at	DNAJC12	56521	NM_0218	-1,90
218264_at	BCCIP	56647	NM_0165	-1,90
226020_s_at	DAB1; ON	115209;	AI927931	-1,89
235587_at	LOC2027	202781	BG40059	-1,89
217764_s_at	RAB31	11031	AF183421	-1,88
226098_at	IFT80	57560	AB03779	-1,88
209906_at	C3AR1	719	U62027	-1,88
218986_s_at	FLJ20035	55601	NM_0176	-1,88
206529_x_at	SLC26A4	5172	NM_0004	-1,88
203456_at	PRAF2	11230	NM_0072	-1,87
35666_at	SEMA3F	6405	U38276	-1,87
235990_at	n/a	n/a	BF43130	-1,87
1552256_a_at	SCARB1	949	NM_0055	-1,87
203790_s_at	HRSP12	10247	N54448	-1,86
224666_at	NSMCE1	197370	AF161451	-1,86
223276_at	MST150	85027	AF31341	-1,86
227452_at	n/a	n/a	AI832118	-1,86
236462_at	n/a	n/a	AA74231	-1,86
1559921_at	PECAM1	5175	AW13891	-1,86
217762_s_at	RAB31	11031	BE78988	-1,86
203897_at	LYRM1	57149	BE96344	-1,85
202772_at	HMGCL	3155	NM_0001	-1,85
226731_at	PELO	53918	AA15687	-1,85
38241_at	BTN3A3	10384	U90548	-1,85
219612_s_at	FGG	2266	NM_0005	-1,84
220992_s_at	C1orf25	81627	NM_0309	-1,84
236520_at	n/a	n/a	AW97238	-1,83
233090_at	n/a	n/a	AU14414	-1,83
202887_s_at	DDIT4	54541	NM_0190	-1,83
230799_at	LOC1508	150837	W72564	-1,83
239903_at	TPBG	7162	AA56585	-1,83
208651_x_at	CD24	934	M58664	-1,83
209522_s_at	CRAT	1384	BC00072	-1,83
203186_s_at	S100A4	6275	NM_0029	-1,83
212613_at	BTN3A2	11118	AI991252	-1,83
209846_s_at	BTN3A2	11118	BC00283	-1,82
228763_at	CHMP4A /	145553 //	AI813313	-1,82
209774_x_at	CXCL2	2920	M57731	-1,82
226093_at	DCP1B	196513	AW20408	-1,82
205119_s_at	FPR1	2357	NM_0020	-1,82
218086_at	NPDC1	56654	NM_0153	-1,81
226151_x_at	CRYZL1	9946	AK00129	-1,81
224448_s_at	C6orf125	84300	BC00600	-1,81
203227_s_at	TSPAN31	6302	NM_0059	-1,81

229860_x_at	LOC4011:	401115	AI341602	-1,81
225059_at	AGTRAP	57085	BE87556:	-1,81
233037_at	n/a	n/a	AF13885:	-1,81
223092_at	ANKH	56172	AA85494:	-1,80
231055_at	n/a	n/a	BF432941	-1,80
219155_at	PITPNC1	26207	NM_0124	-1,80
209916_at	DHTKD1	55526	BC00247:	-1,80
225344_at	NCOA7	135112	AL03568:	-1,80
221430_s_at	RNF146	81847	NM_0309	-1,80
235723_at	BNC2	54796	AA84324:	-1,80
224566_at	TncRNA	283131	AI042152	-1,80
237839_at	n/a	n/a	BF43397:	-1,79
225645_at	EHF	26298	AI763378	-1,79
231175_at	C6orf65	221336	N48613	-1,79
213463_s_at	KIAA0974	317662	AW30050	-1,79
229810_at	n/a	n/a	AI796536	-1,78
218764_at	PRKCH	5583	NM_0240	-1,78
204375_at	CLSTN3	9746	NM_0147	-1,78
236186_x_at	IL17RE	132014	AW00325	-1,78
235795_at	PAX6	5080	AW08823	-1,78
201147_s_at	TIMP3	7078	BF34708:	-1,78
216110_x_at	n/a	n/a	AU14701:	-1,77
220301_at	CCDC102	79839	NM_0247	-1,77
57715_at	FAM26B	51063	W72694	-1,77
213110_s_at	COL4A5	1287	AW05217	-1,77
205660_at	OASL	8638	NM_0037	-1,76
224565_at	TncRNA	283131	BE67551(	-1,76
223384_s_at	TRIM4	89122	BE50146:	-1,76
209140_x_at	HLA-B	3106	L42024	-1,76
1569540_at	n/a	n/a	BC03595:	-1,76
207018_s_at	RAB27B	5874	NM_0041	-1,76
211329_x_at	HFE	3077	AF11526:	-1,75
209834_at	CHST3	9469	AB01791:	-1,75
203108_at	GPRC5A	9052	NM_0039	-1,75
1556436_at	C8orf50	340414	BC04320:	-1,74
227988_s_at	VPS13A	23230	AW62901	-1,74
202888_s_at	ANPEP	290	NM_0011	-1,74
32042_at	ENOX2	10495	S72904	-1,74
229512_at	n/a	n/a	BE46433:	-1,74
224821_at	ABHD14B	84836	AL52020(	-1,73
204977_at	DDX10	1662	NM_0043	-1,73
225457_s_at	LOC2584!	25845	BF52864(	-1,73
235821_at	n/a	n/a	AI917494	-1,73
218177_at	CHMP1B	57132	AA29350:	-1,73
229441_at	PRSS23	11098	AI569872	-1,73
