# Supplementary material for: Identification of target genes for wild type and truncated HMGA2 in mesenchymal stem-like cells
Source: BMC Cancer. 2010 Jun 25;10:329. doi: 10.1186/1471-2407-10-329 (PMC2912264; doi:10.1186/1471-2407-10-329)
Supplement: Additional file 3 — Additional Table S3 Predicted NF-κB recognition sequences in affected genes. [file 1471-2407-10-329-S3.DOC]

**Supplementary table 2 Predicted NFkB recognition sequences in affected genes**

| Gene | Potential NF-κB sites | References |
| --- | --- | --- |
| IL1B | ^-295^GGGAAAATCC | Functional binding site for NF-κB (Hiscott et al., 1993) |
| IL1RN | ^-93^GGGTATTTCC | Functional binding site for NF-κB (Smith et al., 1994) |
| IL11 | ^-688^GGGGTCTCCC  ^-677^GGGTCTCCC | Functional binding sites for NF-κB (Bitko et al., 1997) |
| IL15 | ^-212^GGGCTCCTC | Functional binding site for NF-κB (Azimi et al., 1998) |
| IL6 | ^-75^GGGATTTTCC | Functional binding site for NF-κB (Kanabiran et al., 1997) |
| IL8/CXCL8 | ^-81^TGGAATTTCC | Functional binding site for NF-κB (Kunsch et al., 1994) |
| CXCL1 | ^-79^GGGAATTTCC | Functional binding site for NF-κB and HMGA1 that recognizing the AT-rich sequence within (Wood et al., 1995) |
| CXCL2 | ^-79^GGGAATTTCC | Putative binding site for NF-κB due to similarity (Widmer et al., 1993) |
| CXCL3 | ^-77^GGGAATTTCC | Putative binding site for NF-κB due to similarity (Widmer et al., 1993) |
| CXCL6 | ^-107^GGGAATTTCC | Putative binding site for NF-κB (Zhu et al., 2006) |
| LIF | ^-85^GGGGATCCCG | Predicted by MatInspector (this work). |
| miR-146a | ^-442^GGGATTTCCC  ^-119^GGGACTTTCC | Functional binding binding sites for NF-κB (Konstantin et al., 2006) |
| NFKBIA | ^-319^GGAAACCC  ^-225^GGGAGGACT  ^-63^GGAAATTCCC | Functional binding sites for NF-κB (Hinz et al., 2002) |
| THBS2 | ^-2005^GGGACACCC  ^-480^GGGGATCC | Putative binding sites for NF-κB (Adolph et al., 1997) |
| IGFBP1 | ^-499^GGGCTTTTC | Predicted by MatInspector (this work). |
| PTGES | ^-541^GAAAAGTCC | Putative binding site for NF-κB (Acherman et al., 2008) |
| SERPINB1 | ^-529^GGGATTGGCC  ^-828^GGGAATTTCAC | Predicted by MatInspector (this work). |
| S100A4 | ^+294^GGGTTTTTCC | Functional binding site for NF-κB and κ recognition component (KRC) in intronic enhancer (Cohn et al., 2001) |
| JUNB | ^+2055^GGGCTTTCC  ^+2143^GGGAATCCC  ^+2198^GGGATTTTCC | Putative binding site for NF-κB in downstream enhancer region (Brown et al., 1995) |
| IFI44L | ^-2437^GTGAATTTCC  ^-2263^GGGATTTTCG | Predicted by MatInspector (this work). |
