# Supplementary material for: Identification of target genes for wild type and truncated HMGA2 in mesenchymal stem-like cells
Source: BMC Cancer. 2010 Jun 25;10:329. doi: 10.1186/1471-2407-10-329 (PMC2912264; doi:10.1186/1471-2407-10-329)
Supplement: Additional file 4 — Additional Table S4 Predicted STAT2 recognitions sequences in affected genes. [file 1471-2407-10-329-S4.DOC]

**Supplementary table 3 Predicted STAT2 recognitions sequences in affected genes**

| Gene | Potential Stat2 sites | References |
| --- | --- | --- |
|  | RGGAAANNGAAACT | Consensus ISRE motif |
| IFITM1 | ^-173^AGGAAATAGAAACT  ^-138^GAGAAACTGAAACG | Putative binding sites for ISGF3G due to similarity (Lewin et al, 1991) |
| IFI27 | ^-98^TGGAAAATGAAACC | Functional binding site for ISGF3G (Martensen et al., 2001) |
